# Balancing WNT signalling in early forebrain development: The role of LRP4 as a modulator of LRP6 function

**DOI:** 10.3389/fcell.2023.1173688

**Published:** 2023-04-07

**Authors:** Shuang Geng, Fabian Paul, Izabela Kowalczyk, Sandra Raimundo, Anje Sporbert, Tamrat Meshka Mamo, Annette Hammes

**Affiliations:** ^1^ Neuroscience, Max-Delbrück-Center for Molecular Medicine in the Helmholtz Association (MDC), Berlin, Germany; ^2^ Institute for Biology, Free University of Berlin, Berlin, Germany; ^3^ Advanced Light Microscopy Technology Platform, Max-Delbrück-Center for Molecular Medicine in the Helmholtz Association (MDC), Berlin, Germany

**Keywords:** LRP4, forebrain, development, WNT pathway, genetic modifier, LRP6, neuronal progenitor, neuroepithelium

## Abstract

The specification of the forebrain relies on the precise regulation of WNT/ß-catenin signalling to support neuronal progenitor cell expansion, patterning, and morphogenesis. Imbalances in WNT signalling activity in the early neuroepithelium lead to congenital disorders, such as neural tube defects (NTDs). LDL receptor-related protein (LRP) family members, including the well-studied receptors LRP5 and LRP6, play critical roles in modulating WNT signalling capacity through tightly regulated interactions with their co-receptor Frizzled, WNT ligands, inhibitors and intracellular WNT pathway components. However, little is known about the function of LRP4 as a potential modulator of WNT signalling in the central nervous system. In this study, we investigated the role of LRP4 in the regulation of WNT signalling during early mouse forebrain development. Our results demonstrate that LRP4 can modulate LRP5- and LRP6-mediated WNT signalling in the developing forebrain prior to the onset of neurogenesis at embryonic stage 9.5 and is therefore essential for accurate neural tube morphogenesis. Specifically, LRP4 functions as a genetic modifier for impaired mitotic activity and forebrain hypoplasia, but not for NTDs in LRP6-deficient mutants. *In vivo* and *in vitro* data provide evidence that LRP4 is a key player in fine-tuning WNT signalling capacity and mitotic activity of mouse neuronal progenitors and of human retinal pigment epithelial (hTERT RPE-1) cells. Our data demonstrate the crucial roles of LRP4 and LRP6 in regulating WNT signalling and forebrain development and highlight the need to consider the interaction between different signalling pathways to understand the underlying mechanisms of disease. The findings have significant implications for our mechanistic understanding of how LRPs participate in controlling WNT signalling.

## 1 Introduction

Forebrain development is dependent on the orchestration and integration of various signalling pathways, with the WNT pathway as one of the pivotal players. An intricate balance of WNT signalling is crucial for early forebrain patterning and morphogenesis (Harrison-Uy and Pleasure, 2012; Engelhardt et al., 2022). Impaired WNT signalling leads to severe developmental disorders including neural tube defects (NTDs) (Copp et al., 2003; Wallingford, 2006; Ybot-Gonzalez et al., 2007; Wang et al., 2019; Brown et al., 2020; Zhao et al., 2022). Given that NTDs have a global prevalence of around 19 cases per 10,000 births and are therefore among the most common birth defects (Copp et al., 2003; Harris and Juriloff, 2007; Greene and Copp, 2014; Kakebeen and Niswander, 2021), it is crucial to understand the underlying pathomechanisms.

WNT ligands, such as WNT1 and WNT3a, bind to various types of receptors and co-receptors to transduce signals *via* the canonical β-catenin-dependent and the non-canonical β-catenin-independent pathways. The most prominent non-canonical WNT pathway is the planar cell polarity (PCP) signalling pathway that regulates cytoskeleton dynamics and collective tissue movements crucial for driving neural tube closure processes (Copp et al., 2003; Wallingford, 2006; Ybot-Gonzalez et al., 2007; Wang et al., 2019). In context of neural tube development, the canonical WNT pathway has mostly been implicated in the regulation of regional identity and in balancing proliferation versus differentiation of early neuronal progenitor cells (NPCs) (Harrison-Uy and Pleasure, 2012; Engelhardt et al., 2022). However, there are still many open questions concerning the role of the non-canonical and canonical WNT pathway and their crosstalk in early forebrain development (Da Silva et al., 2021). In particular, the question of how canonical WNT signalling controls the balance between neuronal progenitor cell (NPC) proliferation and differentiation in a temporal and spatial context has not been fully resolved.

The low-density-lipoprotein receptor-related proteins LRP5 and LRP6 are co-receptors for Frizzled (FZD) and mediate canonical ß-catenin dependent WNT signalling (Mao et al., 2001; Ren et al., 2021). Both receptors are closely related and share a high degree of similarity regarding their structure and amino acid sequence. LRP5 and LRP6 are co-expressed during embryonic development in several organs and tissues (Pinson et al., 2000; Houston and Wylie, 2002; He et al., 2004). Gene targeting experiments revealed that LRP5 and LRP6 play distinct roles during development, but there is also functional redundancy between these receptors, since double null mutants show early embryonic lethality before mid-gestation (Kelly et al., 2004). *Lrp5* ^*−/−*^ single mutant mice do not show overt defects during embryonic development, they are viable and fertile. However, adult mice with LRP5 deficiency show low bone mass, impaired retinal vascularization, hypercholesterolemia, impaired insulin secretion and impaired mammary development (Kato et al., 2002; Fujino et al., 2003; Lindvall et al., 2006; Ye et al., 2009), phenotypes that are also seen in patients with *LRP5* loss-of function-mutations (Baron and Kneissel, 2013).

*Lrp6* ^*−/−*^ mutant embryos die at birth and show growth retardation with hypoplasia of the developing neocortex and they exhibit neural tube closure defects (Pinson et al., 2000; Tamai et al., 2000; Wehrli et al., 2000; Mao et al., 2001; He et al., 2004; Kokubu et al., 2004; Zhou et al., 2004; Zhou et al., 2006; Zhou et al., 2008; Zhou et al., 2010; Carter et al., 2005; Song et al., 2009; Song et al., 2010; Wang et al., 2016; Alrefaei and Abu-Elmagd, 2022).

The severe developmental defects, caused by LRP6 deficiency, are associated with impaired canonical and non-canonical WNT signalling levels (Gray et al., 2010; 2013; Allache et al., 2014; Zhao et al., 2022). Decreased non-canonical WNT/PCP signalling may contribute to NTDs in hypermorphic *Lrp6* mutants (Bryja et al., 2009; Gray et al., 2013; Allache et al., 2014), while another recent study demonstrated the essential role of LRP6-mediated canonical WNT/β-catenin signalling in the closure of the posterior neural tube (Zhao et al., 2022). These seemingly contradictory results suggest that cranial and spinal NTDs in LRP6-deficient mutants are caused by distinct pathomechanisms. To fully understand these defects, it is crucial to determine whether increased WNT/PCP signalling activity is indeed a cause of cranial NTDs. Further evidence for the important role of LRP6 during neural tube development comes from patients, carrying *LRP6* mutations, who suffer from impaired neural tube development including NTDs (Allache et al., 2014; Lei et al., 2015; Shi et al., 2018).

Compared to LRP5, LRP6 seems to have a more important impact on signalling pathways during embryonic development. However, the molecular mechanisms that determine how LRP6 senses WNT ligands and regulates downstream signalling cascades are not fully understood (Jeong and Jho, 2021). It has not yet been addressed whether other LRP family members such as LRP5 and LRP4 can modulate LRP6 mediated WNT signalling in the developing forebrain. Whereas LRP5 and LRP6 are clearly defined as co-receptors for WNT, studies on LRP4 focused on its function as an Agrin receptor at the neuromuscular junction. LRP4 binds Agrin which is required for MUSK (Muscle-Specific Kinase) phosphorylation and formation of the neuromuscular junction (Weatherbee et al., 2006; Kim et al., 2008; Zhang et al., 2008; Wu et al., 2012; Yumoto et al., 2012; Barik et al., 2014). Thus, impaired signalling from motor neuron synapses might be the cause of the perinatal lethality in LRP4 loss-of-function mice, due to respiratory failure. Since its initial implication in neuromuscular junction formation and maintenance, several studies have demonstrated the involvement of LRP4 in various neurodevelopmental processes and functions, including peripheral nerve regeneration (Gribble et al., 2018), central nervous system development (Karakatsani et al., 2017; Mosca et al., 2017; Handara et al., 2019; DePew and Mosca, 2021; Yan et al., 2022), cognitive function and plasticity (Gomez et al., 2014; Pohlkamp et al., 2015), and adult hippocampal neurogenesis (Zhang et al., 2019). The significance of LRP4 in nervous system function is further highlighted by its association with several human neurodegenerative diseases, including myasthenia gravis (Kalb et al., 2002; Higuchi et al., 2011; Pevzner et al., 2012; Shen et al., 2013; Tsivgoulis et al., 2014; Chung et al., 2023), amyotrophic lateral sclerosis (ALS) (Tzartos et al., 2014) and Alzheimer’s disease (Choi et al., 2013; Zhang et al., 2020). In a recent study LRP4 was identified as a novel regulator of muscle spindle formation and maintenance in adult and aged mice (Cao et al., 2023) and LRP4 mediates bone homeostasis and mechanotransduction through interaction with sclerostin (Bullock et al., 2019; Choi and Robling, 2021). In contrast to patients with LRP5-deficiency, who suffer from low bone mass, *LRP4* loss-of-function mutations are associated with sclerosteosis 2, characterized by overgrowth of bone mass. (Bukowska-Olech et al., 2020). Deficiency for LRP4 in mice also causes impaired limb formation and polysyndactyly likely due to abnormal WNT signalling in the apical ectodermal ridge (Weatherbee et al., 2006). Furthermore, loss of LRP4 leads to renal agenesis, impaired tooth development and aberrant mammary placode formation (Ohazama et al., 2008; Ohazama et al., 2010; Ahn et al., 2010; Ahn et al., 2013; Ahn et al., 2017). Mutations within the human *LRP4* gene cause kidney malformations and complex syndactyly, which is also referred to as Cenani-Lenz syndrome (Li et al., 2010; Khan et al., 2022). A recent study suggested a role of LRP4 in vascular biology. The authors identified LRP4 as a co-receptor for integrin α_V_β_3_, binding the Von Willebrand factor and inducing human vascular smooth muscle cell proliferation (Lagrange et al., 2022).

Various studies have shown that LRP4 plays an important role in the WNT/β-catenin signalling pathway, although the mechanisms whereby LRP4 modulates WNT signalling are less well understood. The extracellular ligand binding domain of LRP4 shares structural elements with LRP5 and LRP6. The intracellular domain of LRP4 however lacks motifs that are essential for WNT co-receptor function and which are present in LRP5 and LRP6. Therefore it was proposed that LRP4 can act as a negative regulator of WNT signalling (Herz and Bock, 2002; Willnow et al., 2012). Further evidence for this hypothesis comes from cell culture showing that overexpressing *Lrp4* decreased canonical WNT signalling activity (Johnson et al., 2005; Li et al., 2010). The extracellular domain of LRP4 can bind the WNT antagonists DKK (Dickkopf-related protein 1) and WISE (SOSTDC-1 = Sclerostin domain-containing protein 1) (Ohazama et al., 2008; Choi et al., 2009; Karner et al., 2010). Elegant mouse genetic studies provided evidence that LRP4 modulates WNT/ß-catenin signalling activity during mammary gland and tooth development through its interplay with WISE and also independently of WISE (Ahn et al., 2013; Ahn et al., 2017). However, it still remained unresolved whether LRP4 plays a role in canonical WNT signalling as a negative regulator antagonizing LRP6-mediated pathway activation also in other ectodermal tissue such as the developing neural tube. To better understand the function of the WNT pathway in the developing forebrain and to test whether LRP4 can balance WNT signalling, we analysed *Lrp6* ^−/−^ mutant mice as a model of diminished canonical WNT signalling and *Lrp4* ^*−/−*^; *Lrp6* ^*−/−*^ double mutants to investigate LRP4’s potential physiologic *in vivo* functions in modulating WNT signalling during early forebrain development.

## 2 Materials and methods

### 2.1 Animals

The ENU induced *Lrp4* ^*mitt*^ mutant mouse line was generated the laboratory of Lee Niswander (Weatherbee et al., 2006) and was kindly provided by Scott Weatherbee and Robert Krumlauf.

In this study, *Lrp4* ^*mitt*^ heterozygotes are referred to as *Lrp4* ^*+/−*^ and *Lrp4* ^*mitt*^ homozygotes with a complete loss of LRP4 are referred to as *Lrp4* ^*−/−*^. The *Lrp5* ^*tm1Lex*^ mouse line was generated by gene targeting in the laboratory of Matthew Warman and Bart Williams, who generously provided this mouse line for this study (Holmen et al., 2004). In this study *Lrp5* ^*tm1Lex*^ mice are referred to as *Lrp5* ^*+/−*^ (heterozygotes) and *Lrp5* ^*−/−*^ mice (homozygotes), respectively. The *Lrp6* ^*Gt(Ex187)Byg*^ functional null mouse line was created by William Skarnes (Pinson et al., 2000) and obtained from BayGenomics *via* Jackson Laboratories. In this study heterozygous and homozygous mutants are referred to as *Lrp6* ^*+/−*^ and *Lrp6* ^*−/−*^, respectively.

To generate *Lrp4* ^*mitt*^; *Lrp5* ^*tm1Lex*^ functional null double mutant embryos, *Lrp4* ^*+/−*^; *Lrp5* ^*+/−*^ x *Lrp4* ^*+/−*^; *Lrp5* ^*+/−*^ or *Lrp4* ^*+/−*^; *Lrp5* ^*+/−*^ x *Lrp4* ^*+/−*^; *Lrp5* ^*−/−*^ adult mice were bred for timed mating. *Lrp4* ^*mitt*^; *Lrp5* ^*tm1Lex*^ functional null double mutants are referred to as *Lrp4* ^*−/−*^; *Lrp5* ^*−/−*^ embryos.

*Lrp4* ^*mitt*^; *Lrp6* ^*Gt(Ex187)Byg*^ functional null double mutant embryos were generated by combining two *Lrp4* ^*+/−*^; *Lrp6* ^*+/−*^ adult mice in timed mating. *Lrp4* ^*mitt*^; *Lrp6* ^*Gt(Ex187)Byg*^ functional null double mutants are referred to as *Lrp4* ^*−/−*^; *Lrp6* ^*−/−*^ embryos.

The *Tg(TCF/Lef1-HIST1H2BB/EGFP)61Hadj/J* transgenic reporter mouse line was created by Anna-Katerina Hadjantonakis and was obtained from the Jackson Laboratories (MGI:4881498, Common Name: *TCF/Lef:H2B/GFP*). *TCF/Lef:H2B-GFP* transgenic mice express an H2B-EGFP fusion protein under the control of six copies of a T cell specific transcription factor/lymphoid enhancer-binding factor 1 (TCF/Lef1) response element and a heat shock protein 1B (*Hspa1b*) minimal promoter (Ferrer-Vaquer et al., 2010). In this study, the *TCF/Lef:H2B-GFP* transgenic mouse line was used to visualize WNT/ß-catenin-signalling in neuroepithelial cells of the investigated *Lrp4,* and *Lrp6* mouse models. Mice that carry one allele of the *TCF/Lef:H2B-GFP* reporter cassette are referred to as *Gfp* ^*+/−*^ (e.g., *Lrp4* ^*+/−*^; *Gfp* ^*+/−*^).

All mice were crossed onto and kept on a pure C57BL/6N background.

Experiments involving animals were performed according to institutional guidelines following approval by local authorities (X9005/12 and X9001/21).

### 2.2 Genotyping

*Lrp6* mutant mice were genotyped by PCR and by X-gal staining intensity of yolk sacs according to the protocol by Pinson and colleagues (Pinson et al., 2000). Homozygous versus heterozygous *Lrp6* ^*Gt(Ex187)Byg*^ embryos were identified by X-gal staining of the yolk sacs. Yolk sacs were collected and quickly washed in cold 1x PBS. Then the yolk sacs were transferred to X-gal washing buffer and washed for 10 min, shaking at 4°C. Subsequently, the yolk sacs were incubated in X-gal staining solution (1.25 mL 200 mM potassium ferrocyanide solution, 1.25 mL 200 mM potassium ferricyanide solution, 45.8 mL X-gal washing buffer, 1.2 mL X-gal substrate—40 mg X-gal/1 mL DMF) at 37°C. After 15–30 min, the staining intensity was sufficient to discriminate between heterozygous and homozygous samples. Yolk sacs were transferred to X-gal washing buffer (500 µL of Igepal Ca-30, 0.25 mL of 10% deoxycholate solution, 500 mL 1x PBS) to stop the staining process.

The following primer pairs were used for genotyping: Name of primer sequence and sequence (5′ to 3’): *Lrp4-forward*: GGT GAG GAG AAC TGC AAT GT, *Lpr4-reverse*: TGA GTC AAG GTC ACA CCC ATC.

In this study, restriction enzyme HpyCH4V (BioLabs, R0620L) was used to digest the amplified products from the *Lrp4* genotyping PCR. PCR products from the wild-type allele were digested whereas the PCR product from the mutant did not contain the restriction site.

*Lrp5_neo_forward*: GCC TTC TAT CGC CTT CTT GAC, *Lrp5_gen_forward*: AAA CTG TGA CAG GCT GTG GGA AGT, *Lrp5_gen_reverse*: GCC GCA CAC ACC ACC AAA CTA TAA.

*Lrp6 (beta-geo) forward*: CAA ATG GCG ATT ACC GTT GA, *Lrp6 (beta-geo) reverse*: TGC CCA GTC ATA GCC GAA TA.

*TCFgfp-forward (Hadj_F)*: ACA ACA AGC GCT CGA CCA TCA C, *TCFgfp reverse (Hadj_R)*: AGT CGA TGC CCT TCA GCT CGA T.

### 2.3 *In situ* hybridization (ISH) on whole mount samples and cryosections

Whole-mount *in situ* hybridization was carried out as described previously (Hammes et al., 2001). *In situ* hybridization on sections was performed as described previously (Jensen and Wallace, 1997), except that the signal was enhanced by performing the colour reaction in the presence of 10% polyvinyl alcohol (Sigma Aldrich, P8136). Probe synthesis was conducted with the components of the DIG RNA labelling kit (Roche, 11277073910).

Images of embryos were taken using a Leica MZ 10F stereomicroscope (Leica LAS V4.9 imaging software). Images were processed in ImageJ to isolate specimen and adjust the background colour.

Primers used for cloning templates for *in situ* riboprobes: *Lrp4* ISH probe primer forward: TAC CAT CGA AGC ATC TCG GC, reverse: TTC GTG TTT CCA GCC TGT GT; *Lrp5* ISH probe primer forward: ATG CCG GCG GAG TGA AG, reverse: GAG TAG AAA GGC TCC CTC GG.

*Lef1* riboprobe kindly provided by Thomas Willnow and Walter Birchmeier, MDC, Berlin, Germany.

### 2.4 X-gal staining

After fixation of the embryos in 4%PFA, the samples were washed in PBS and transferred to X-Gal washing buffer. Then embryos were submerged in X-Gal staining solution and stained at 37°C or RT. Subsequently, staining process was stopped by transferring the embryos to X-Gal washing buffer.

### 2.5 Real-time quantitative reverse transcription PCR (real-time qRT-PCR)

RNA from hTERT RPE-1 cells was extracted using TRIzol™ Reagent (Thermo Fisher, 15596018). cDNA was synthesized by high-capacity RNA-to-cDNA™ kit (Applied Biosystems, 4387406). Quantitative PCR was performed using TaqMan™ Universal PCR Master Mix (Applied Biosystem, 4369016) with the BioRad CFX384 Real Time System used on a BioRad C1000 Touch Thermal Cycler. The following TaqMan probes (Life Technologies; *Lrp4*: Hs00391006_m1, *Gapdh*: Hs99999905_m1) were used to detect *Lrp4* and *Gapdh* expression, respectively.

The expression levels of *Lrp4* were normalized to *Gapdh* expression. Transcript levels relative to *Gapdh* were calculated using the deltaCt method. Data were analysed in GraphPad Prism 7 using one-way ANOVA.

### 2.6 Immunohistochemistry

Standard immunofluorescence was performed on cryo-sections. PFA fixed embryos were infiltrated with 15% and 30% sucrose in PBS for up to 24 h depending on the stage, embedded in OCT (Tissue-Tek, Sakura Finetek, sa-4583) and cut into 10 μm coronal sections. Cryosections were stored at −20°C until further processing. Frozen sections were removed from −20°C and air dried for 1 h. Slides were transferred to a Coplin jar and washed in PBS + 0.1% Triton-X100 (PBTr) for 5 times, 7 min each. Subsequently, the solution was replaced by PBTr with 10% goat serum and 1%BSA and the slides incubated in this blocking solution for 1 h. Subsequently, the sections were incubated with primary antibody at 4°C overnight. The next day, the primary antibody was discarded, and the sections were washed in PBTr for 7 min, 5 times each. The slides were incubated with secondary antibody and DAPI at RT in the dark for 1 h. Subsequently, the slides were transferred to PBTr and washed for 5 × 7 min at RT avoiding light. In the next step, the sections were quickly washed in water and mounted with fluorescent mounting medium (Dako Fluorescence Mounting Medium (Agilent, S302380-2). The slides were dried in a hood for 3–4 h and stored at 4°C to minimize fading of the fluorophores.

Primary antibodies and final concentration: Cyclin D1, rabbit, Abcam, ab16663, 1:100; Phospho-Histone H3 (pHH3), mouse, Invitrogen, # MA5-15220, 1:250; MPM-2, mouse, Millipore, 05–368, 1:1,500; SOX2, rabbit, Abcam, ab97959, 1:100; GFP, chicken, Abcam, ab13970, IHC—1:200; DAPI, Invitrogen, 62248, 1:1,000; cleaved-Caspase-3, rabbit Cell Signaling CST #9661, 1:1,000.

Secondary antibodies and final concentration: Donkey anti-mouse Alexa Fluor 488, Abcam, ab150109, 1:500; Donkey anti-rabbit Alexa 555, Abcam, ab150074, 1:500; Donkey anti-chicken Alexa 488 Abcam, 1:500.

### 2.7 Cell culture and transfection with siRNA for gene silencing

Human RPE cell line hTERT-RPE1 (ATCC^®^ CRL4000™) was purchased from the American Type Culture Collection (ATCC, Lot #70021355). Cells were maintained in DMEM: F12 Medium (ATCC^®^ 302006™) with 10% Fetal Bovine Serum (FCS, PAN-Biotech GmbH, Cat. #P40-37500) containing 0.01 mg/mL hygromycin B (Santa Cruz, Cat. #sc-506168).

LRP4, and LRP6 silencing was achieved by siRNA transfection. hTERT RPE-1 cells were transfected using Lipofectamine™ RNAiMAX (Thermo Fisher, Cat. #13778–150). The following siRNA reagents were used at final concentration of 10 pmol: Silencer^®^ Select *LRP4* siRNA, Ambion—Thermo Fisher Scientific, cat# 4392420, ID s8289; Silencer^®^Select *LRP6* siRNA, Ambion - Thermo Fisher Scientific, cat# 4390824, ID s8290; Negative control siRNA, Ambion - Thermo Fisher Scientific, cat# 4390847). Three technical replicates were done for each experiment. Experiments were repeated at least three times.

### 2.8 Western blot analysis

Cells were lysed in the RIPA lysis buffer [20 mM Tris-HCl (pH 7.5), 150 mM NaCl, 1 mM Na2EDTA, 1 mM EGTA, 1% NP-40, 1% sodium deoxycholate and 1 mM AEBSF]. Equal amounts of samples were subjected to a Tris-Glycine Gel (Invitrogen, XP0012C) in a Mini Gel Tank (Invitrogen, A25977). The resolved proteins were transferred onto a nitrocellulose membrane (Amersham Protran 0.2 μm, 10600006) using a wet electroblotting system (Bio-Rad Mini Protean II Cell) followed by immunoblotting. 5% non-fat dry milk in 1× TBS-T (0.1% Tween-20) was used for blocking at room temperature for 1 h.

Primary antibodies were applied overnight at 4°C as follows: LRP6, rabbit, Abcam, ab134146, 1:1,000; Cyclin D1, rabbit, Abcam, ab16663, 1:2,500; alpha-Tubulin, mouse, Merck Millipore, CP06, 1:10000; HSP90, rabbit, Cell Signaling Technology, #4874S, 1:1,000; GAPDH, mouse, Santa Cruz biotechnology, sc-32233, 1:10000.

Secondary antibodies: Goat anti-mouse IgG (HRP), Abcam, ab97265, 1:10000; Goat anti-rabbit IgG (HRP) Abcam, ab6721, 1:10000.

Signals were detected by SuperSignal West Dura (Life Technologies, 34075) with an Optimax 2010 X-Ray Film Processor (PROTECT) or using the BioRad ChemiDoc MP Imaging System. The results were quantified using ImageJ, with one-way ANOVA as a statistical analysis.

### 2.9 Immunocytochemistry

Cells were split and seeded into a 24 well plate coated with coverslips (Paul Marienfeld, 0111520) in a final concentration 4 × 10^4^ cells/ml. After 48 h of transfection with siRNA as describes above, cells were quickly washed with cold 1x PBS (1mL/well) and fixed with 4% PFA (500µL/well) for 15 min at RT. Cells were permeabilized by 1x PBS with 0.25% Triton X-100 (500μL/well) at RT for 20 min. Next, the cells were blocked with 10% donkey serum in 1x PBS-Triton 0.25% (500μL/well) for 1 h at RT.

Primary antibodies were diluted in 0.25% PBS-Triton and dropped to a parafilm as 80μL/drop. Then the coverslips were taken out from plate and put on the drops with cell facing down. The cells were incubated with primary antibodies for 1 h at RT. After that, the coverslips were placed back on the 24 well plate and the cells were washed with 0.25% Triton X-100 for 3 × 10 min. Secondary antibodies were diluted 1:500 and applied in the same way as primary antibodies. Cells were incubated with secondary antibodies in the dark for 1 h at RT. Subsequently, cells were washed with 1x PBS 0.25% Triton X-100 for 3 × 10 min in a 24 well plate. Afterwards, the coverslips were mounted with DAKO.

### 2.10 Confocal microscopy image acquisition

Image acquisitions of tissue sections were carried out using either a Leica SPE or Leica TCS SP8 confocal microscope using a HC Pl Apo 20× NA 0.75 MultiIMM and HC Pl Apo 63× NA 1.3 oil immersion objective. All samples that were compared to each other either for qualitative or quantitative analysis were imaged under identical settings for laser power, detector and pixel size.

hTERT RPE-1 cells were imaged with Zeiss LSM 700 confocal microscope equipped with a plan-Apo 63 × 1.4 oil immersion objective.

In all samples, Alexa Fluor 488 was excited by a 488 nm laser, detection at 500–550 nm, Alexa Fluor 555 was excited by a 555 nm laser, detection at 570–620 nm, Alexa Fluor 647 was excited by a 633 nm or 647 nm laser, detection at 660–730 nm, and DAPI was excited at 405 nm, detection at 420–450 nm with a pinhole set to 1 AU.

### 2.11 Quantification of immunofluorescence signal intensity

Z-stack images of the coronal sections and for immunocytochemistry on hTERT RPE-1 cells were analysed using ImageJ (Fiji, NIH). For the quantification of fluorescence signals in the neuroepithelium, the full z-stack was used. The region of interest (ROI) was manually outlined and the mean fluorescent intensity was measured with the ROI manager. The average intensity for each sample was used in the final quantification and one-way ANOVA or unpaired *t*-test statistical analysis was performed to assess the significance.

### 2.12 Quantification and statistical analysis

Tests used to analyse the data were carried out using Prism 7 software (GraphPad) and are mentioned in the respective figure legends. Figures were prepared using Inkscape version 1.2.

The standard error of mean (SEM) is provided. The term significant was used if *p* values were below 0.05 (*p* < 0.05). Exact *p* values, n numbers and biological replicates are reported in the figure legends.

## 3 Results

### 3.1 Overlapping and distinct expression patterns for *Lrp4*, *Lrp5* and *Lrp6* in the developing forebrain

In this study we aimed at shedding light on the roles of LRP4, LRP5 and LRP6 in WNT signalling-dependent development of the murine forebrain. The functions of these receptors are unclear especially during early stages of embryonic forebrain development before and around mid-gestation. We first assessed mRNA expression patterns for all three receptors in the anterior neural tube at early developmental stages.

**FIGURE 1:**
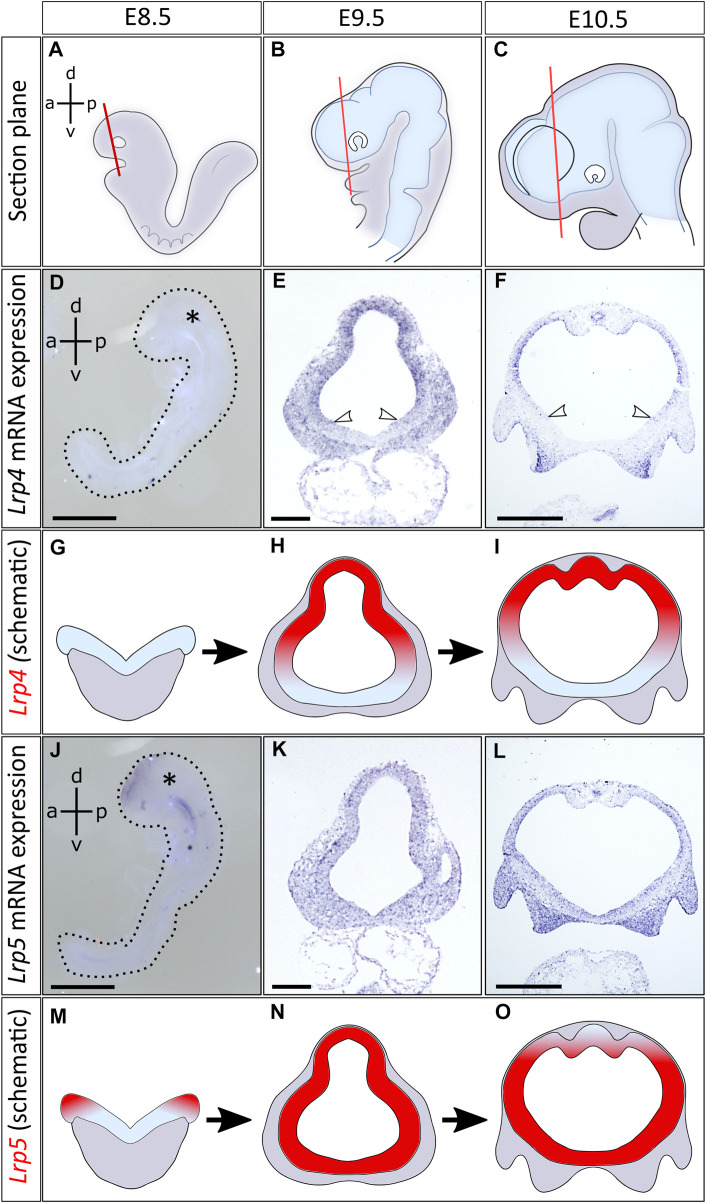
Distinct and overlapping expression of *Lrp4* and *Lrp5* mRNA in the early developing neural tube. **(A–C)** Schematics indicate planes of coronal sections of mouse embryonic forebrains, anterior to the optic cup, between embryonic stages E8.5 and E10.5. Images of further section planes are presented in Supplementary Figure S1. **(D)** *Lrp4* mRNA expression could not be detected by whole mount *in situ* hybridization (ISH) at E8.5 (whole embryo at E8.5, lateral view, asterisk marks the head, dorsoventral- (d-v) and anteroposterior axis (a-p) are indicated, scale bar: 100μm, *n* = 4 embryos). **(E)** At E9.5 *Lrp4* mRNA could be visualized by ISH on coronal sections of the forebrain. *Lrp4* was expressed in the entire dorsal lateral domain of the neural tube including the dorsal midline, while the ventral midline was always void of *Lrp4* transcripts (white arrowheads: ventral border of *Lrp4* expression domain, scale bar: 100μm, *n* = 5 embryos). **(F)** At E10.5 *Lrp4* continued to be expressed in the neuroepithelium showing ISH signals in the dorsolateral but not in the ventral domains of the forebrain (white arrowheads indicating the ventral border of *Lrp4* expression, scale bar: 500μm, *n* = 5 embryos). **(G–I)** Schematics indicating the *Lrp4* transcript distribution (red) on coronal forebrain sections between E8.5 and E10.5. **(J)** *Lrp5* was already expressed at E8.5 in the neural folds. Whole mount ISH shows *Lrp5* transcripts in the neural folds (whole embryo at E8.5, lateral view, asterisk marks the head, dorsoventral (d-v) and anteroposterior axis (a-p) are indicated, scale bar: 100μm, *n* = 4 embryos). **(K)** ISH on coronal sections at E9.5 indicate that *Lrp5* is widely expressed in the neural tube (scale bar: 100μm, *n* = 3 embryos). **(L)** At E10.5, *Lrp5* continued to be expressed in neuroepithelial cells of the entire neural tube. Little signals are seen in the dorsal midline (scale bar: 500μm, *n* = 4 embryos). **(M–O)** Schematics indicating *Lrp5* transcript distribution (red) on coronal forebrain sections between E8.5 and E10.5.

*Lrp4* transcripts were detected in the rostral neural tube starting at E9.5. The receptor was expressed in a broad domain in the dorsolateral region of the forebrain, whereas the ventral midline was always void of *Lrp4* transcripts (Figures 1A–I). This pattern for *Lrp4* expression was seen throughout the entire forebrain region, including the anterior and posterior telencephalon as well as the diencephalon (Supplementary Figures S1A–H). In contrast to *Lrp4*, expression of *Lrp5* could already be detected in E8.5 mouse embryos. *Lrp5* mRNA was first expressed in the dorsal domains of the neural folds **(** Figures 1J, M). At E9.5 and E10.5, *Lrp5* expression profile extended to the entire neural tube with strongest signals in the lateral domain and fainter signals in the dorsal midline (Figures 1K, L, N, O). *Lrp5* expression was seen throughout the telencephalic and diencephalic forebrain regions (Supplementary Figures S1I–P **)**. *Lrp6* was ubiquitously expressed in the neural folds starting from E8.5 (Supplementary Figures S1Q–T).

We could show that from E9.5 onwards, *Lrp4*, *Lrp5* and *Lrp6* gene expression profiles overlapped to a great extent in the neuroepithelium of the developing forebrain, suggesting possible functional interactions. Besides the overlapping expression pattern in the lateral forebrain, *Lrp4* and *Lrp5* also showed distinct domains regarding their signal strength, with *Lrp4* being more prominently expressed in the dorsal midline and *Lrp5* in the ventral forebrain. To dissect common and distinct functions of these LRP candidates during forebrain formation, with a focus on the role of LRP4, and to reveal potential gene interactions, we next generated double null mutants for phenotypic analyses on the forebrain, primarily at embryonic stage E9.5.

### 3.2 *Lrp4* ^*−/−*^ *; Lrp5* ^*−/−*^ double null mutants suffer from impaired neural tube closure and die at embryonic stage E10.5

**FIGURE 2:**
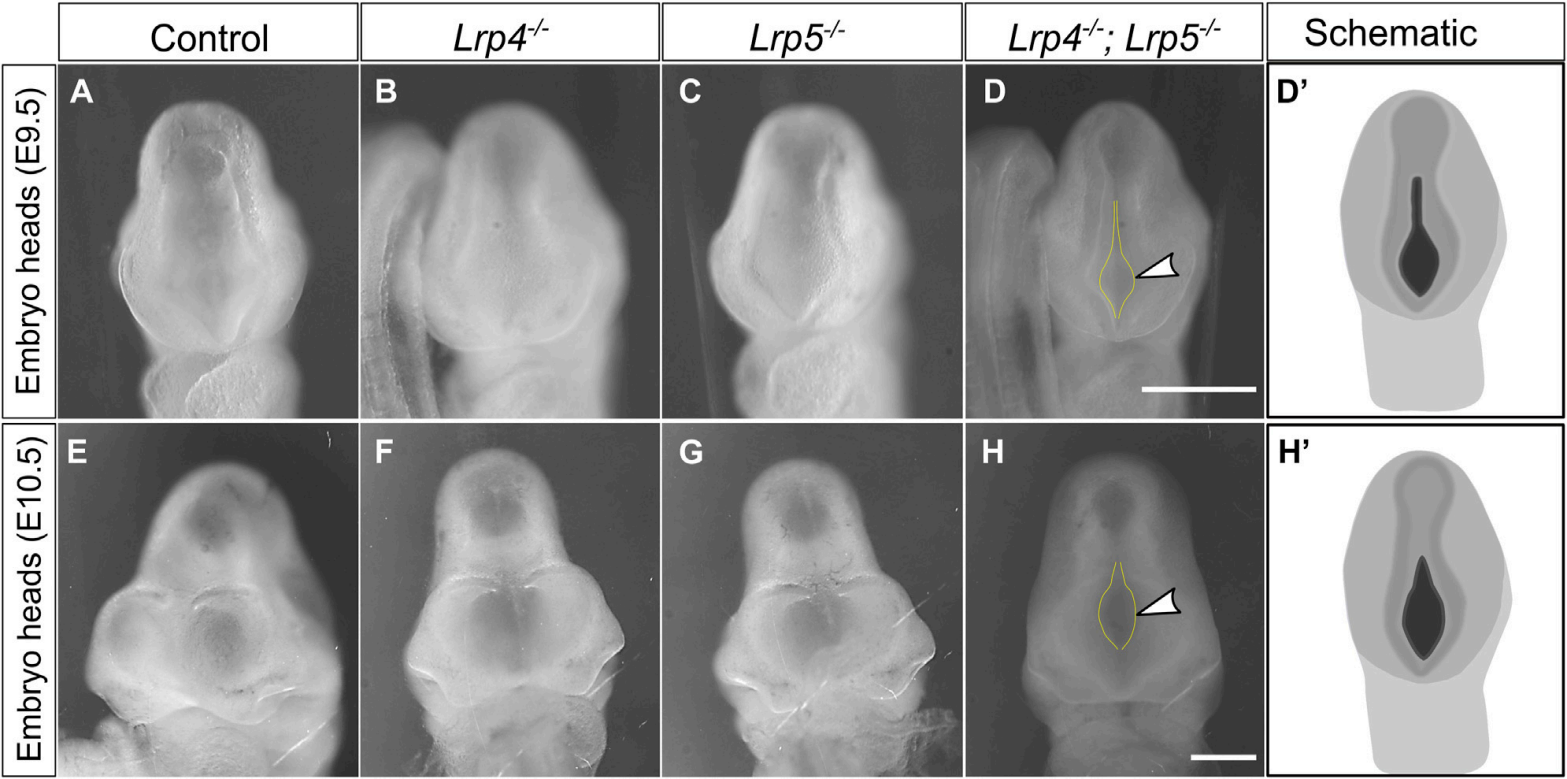
Loss of both, LRP4 and LRP5, leads to impaired cranial neural tube closure. **(A–C)** At E9.0 and E9.5 (somite stages 20–25) all *Lrp4* ^*−/−*^ single mutant mouse embryos **(B)** (*n* = 5), all *Lrp5* ^*−/−*^ mutants **(C)** (*n* = 3) and all wild-type littermate controls **(A)** (*n* = 5) displayed a closed anterior neuropore (ANP). **(D)** In contrast, 88% of all *Lrp4* ^*−/−*^; *Lrp5* ^*−/−*^ double mutant embryos (8 out of 9) had an open anterior neuropore (frontal view of whole embryo heads, dotted yellow lines and arrowhead indicate the open ANP, scale bar: 500 µm); **(D′)** Schematic indicating the open anterior neuropore at E9.5 in black. **(E–G)** At E10.5 wild-type controls **(E)** (*n* = 3), *Lrp4* ^*−/−*^ embryos **(F)** (*n* = 4), and *Lrp5* ^*−/−*^ embryos **(G)** (*n* = 3) displayed normal cross morphology of the forebrain and the ANP was closed (frontal view of whole embryo heads). **(H)** 80% of the *Lrp4* ^*−/−*^; *Lrp5* ^*−/−*^ double mutant embryos showed an open anterior neuropore (dotted line and arrowhead) at E10.5 (4 out of 5 embryos). Scale bar: 500 μm. **(H′)** Schematic indicating the open anterior neuropore at E10.5 in black.

Loss of LRP6 leads to neural tube defects (NTD) as reported before (Gray et al., 2010; 2013; Allache et al., 2014; Zhao et al., 2022), while no such defects have been observed in LRP4 loss-of-function mutants or *Lrp5* ^*−/−*^ mutant embryos. Since *Lrp4* and *Lrp5* are highly expressed during forebrain development, they may have functional redundancy during early forebrain development. In wild-type embryos, the anterior neuropore (ANP) is typically fused by the 20 somite stage (Theiler, 1989). Accordingly, complete closure of the ANP was observed at somite stages 20 and later in all analysed wild-type, *Lrp4* ^*−/−*^, and *Lrp5* ^*−/−*^ single mutant embryos (Figure 2). In contrast, *Lrp4* ^*−/−*^; *Lrp5* ^*−/−*^ double mutant embryos at somite stages 20–35 (embryonic stage 9.0–10.5) still displayed an open ANP closure in 12 out of 14 embryos (Figure 2). These findings suggest that LRP4 and LRP5 are crucial for early forebrain morphogenesis and the precise timing of neural tube closure processes. Interestingly, *Lrp4* ^*−/−*^; *Lrp5* ^*−/−*^ double mutant embryos did not survive beyond E11.5, while *Lrp4* ^*−/−*^ and *Lrp5* ^*−/−*^ single mutants survived throughout embryonic development (Supplementary Figures S2A–D) indicating their functional redundancy during early embryonic development. The cause of embryonic lethality is likely due to cardiovascular defects.

### 3.3 *Lrp4* is a genetic modifier for neuroepithelial hypoplasia phenotypes in *Lrp6* ^*−/−*^ mutants but not for cranial neural tube defects

LRP5 shares a similar molecular structure with LRP6 (Ahn et al., 2011; Joiner et al., 2013) and several studies have reported their functional redundancy during embryonic development (Holmen et al., 2004; Ren et al., 2021). To investigate common and distinct functions of LRP5 and LRP6 in their interplay with LRP4, regarding WNT signalling-related embryogenesis, and to shed light on a potential interaction between LRP4 and LRP6 during forebrain development, we next generated *Lrp4* ^*−/−*^; *Lrp6* ^*−/−*^ double mutant embryos. Unlike *Lrp4* ^*−/−*^; *Lrp5* ^*−/−*^ double mutants, *Lrp4* ^*−/−*^; *Lrp6* ^*−/−*^ embryos survived throughout all embryonic stages (Supplementary Figures S2E, F).

**FIGURE 3:**
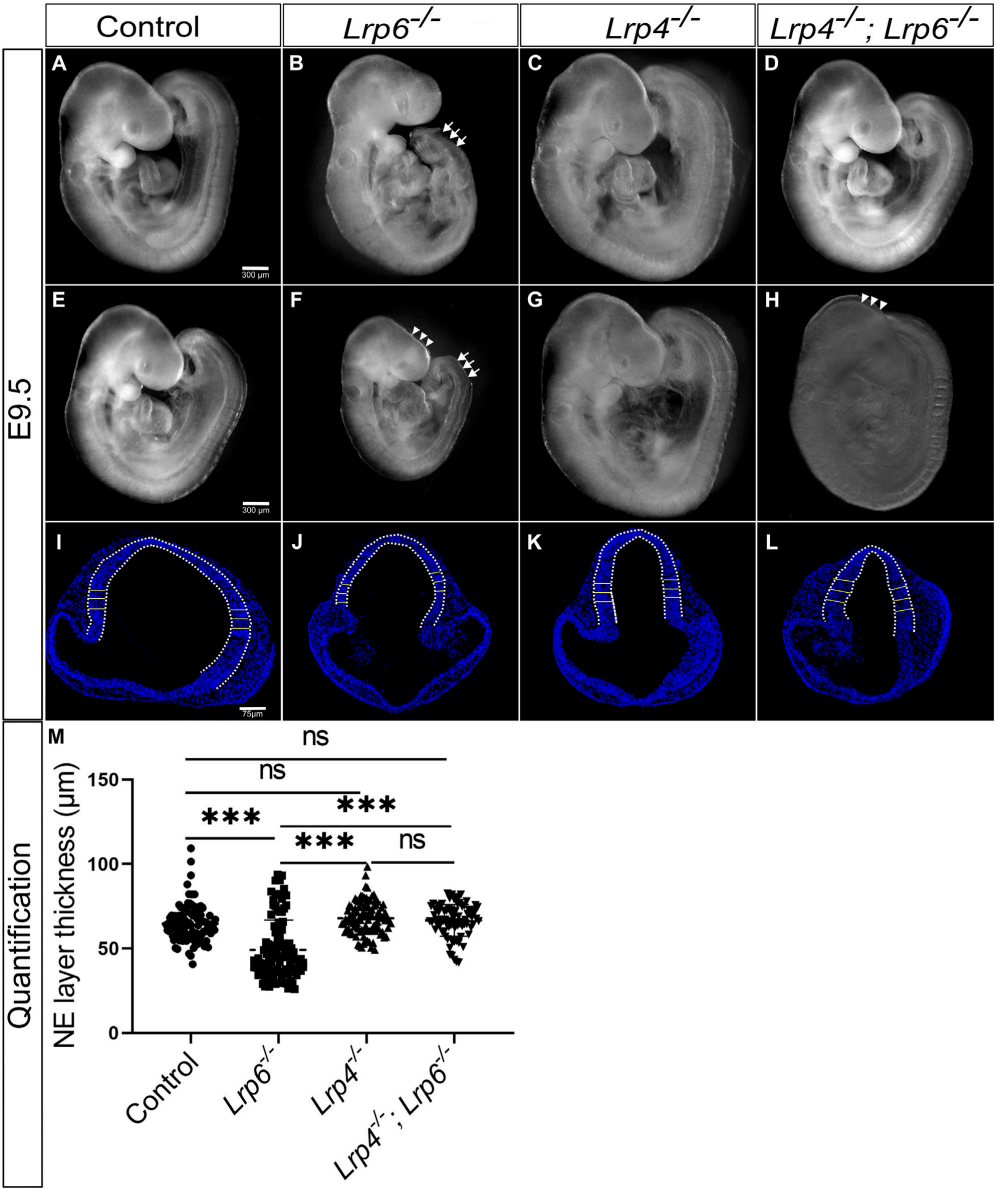
Genetic ablation of LRP4 function in rescues neuroepithelial hypoplasia but not cranial neural tube defects (NTDs) in *Lrp6* ^*−/−*^ mutants. **(A–H)** Lateral views of whole embryos at E9.5. As reported before, the caudal truncation phenotype was fully penetrant in *Lrp6* ^*−/−*^ mutants [**(B, F)**, arrows]. 32% of *Lrp6* ^*−/−*^ mutants (8 out of 25) displayed cranial NTDs [**(F)**, arrowheads] compared to stage matched littermate wild-type controls **(A, E)** and 68% of *Lrp6* ^*−/−*^ mutants (17 out of 25) had a closed cranial neural tube **(B)**. *Lrp4* ^*−/−*^ mutants **(C, G)** showed a cross morphology comparable to wild-type controls and never displayed NTDs. *Lrp4* ^*−/−*^; *Lrp6* ^*−/−*^ double mutants had a less severe caudal truncation **(D, H)**, the cranial NTD however was seen in a similar frequency as in *Lrp6* ^*−/−*^ mutants. 62.5% of the *Lrp4* ^*−/−*^; *Lrp6* ^*−/−*^ mutants (15 out of 24) had a closed anterior neural tube **(D)** whereas 37.5% (9 out of 24) *Lrp4* ^*−/−*^; *Lrp6* ^*−/−*^ mutants suffered from cranial NTDs **(H)**. Scale bars: 300 µm. **(I–L)** Representative images of DAPI stained coronal sections with the bars indicating the thickness of the forebrain neuroepithelium at E9.5 measured along the dorsolateral domain indicated by the dotted line. *Lrp6* ^*−/−*^ mutants **(J)** displayed in average a significantly thinner neuroepithelium compared to embryonic stage-matched wild-type controls **(I)** and *Lrp4* ^*−/−*^ mutants **(K)**, which had normal neuroepithelial morphology comparable to controls. *Lrp4* ^*−/−*^; *Lrp6* ^*−/−*^ double mutants **(L)** showed a rescue of neuroepithelium thickness compared to *Lrp6* ^*−/−*^ single mutants and had a neuroepithelial morphology comparable to controls. Scale bar: 75 µm. **(M)** The graph shows individual points, representing the individual measurements of the forebrain neuroepithelium thickness. Three regions from the lateral domain, as indicated by the horizontal lines, were measured from each section. For each sample, 5 to 15 sections were examined; *n* = 4 embryos for controls, *n* = 3 *Lrp6* ^*−/−*^ mutants, *n* = 3 *Lrp4* ^*−/−*^ mutants, *n* = 4 *Lrp4* ^*−/−*^; *Lrp6* ^*−/−*^ double mutants; statistics: one-way ANOVA; NE: neuroepithelium.

Consistent with previously published data, reporting 30% penetrance of cranial neural tube closure defects causing exencephaly phenotypes in *Lrp6* ^*−/−*^ mutant mouse embryos (Carter et al., 2005; Gray et al., 2010; Gray et al., 2013), we observed similar rates of anterior NTDs in *Lrp6* ^*−/−*^ single mutants (Figures 3B, F). *Lrp4* ^*−/−*^ single mutants never displayed NTDs (Figures 3C, G), also consistent with previously published data on *Lrp4* mutants (Weatherbee et al., 2006). Control wild-type embryos are shown in (Figures 3A, E). *Lrp4* ^*−/−*^ *; Lrp6* ^*−/−*^ double mutants exhibited similar rates of NTDs as *Lrp6* ^*−/−*^ single mutants (Figures 3D, H), indicating that *Lrp4* is not a genetic modifier for cranial NTDs in *Lrp6* mutants with defects in the non-canonical WNT pathway. However, a clear difference in the neuroepithelial thickness was observed in *Lrp4* ^*−/−*^; *Lrp6* ^*−/−*^ double mutants compared to *Lrp6* ^*−/−*^ single mutants at E9.5 (Figures 3I–M). Consistent with previous results (Gray et al., 2010; Gray et al., 2013) *Lrp6* ^*−/−*^ mutants suffered from neuroepithelial hypoplasia (Figures 3J, M), which was not caused by increased apoptosis (Supplementary Figure S3). In *Lrp4* ^*−/−*^; *Lrp6* ^*−/−*^ double mutants, genetic ablation of *Lrp4* partially rescued neuroepithelial hypoplasia in *Lrp6* ^*−/−*^ mutants (Figures 3L, M). We thus conclude that *Lrp4* can be a genetic modifier for the growth retardation and forebrain hypoplasia phenotypes caused by *Lrp6* loss-of-function mutations but not for the neural tube closure defects.

### 3.4 Decreased proliferation of forebrain neuronal precursors in *Lrp6* ^*−/−*^ mutants is reversed by genetic inactivation of LRP4

Defects in neural tube patterning, such as the thinning of the neuroepithelial layer due to disruption of the pseudostratification, are often associated with aberrant progenitor cell proliferation. Early symmetric divisions in the pseudostratified neuroepithelium are responsible for the expansion of neuroepithelial cells before neurogenesis. Therefore, we next examined whether self-renewal of neuroepithelial cells was altered in the forebrain of *Lrp4* ^*−/−*^, *Lrp6* ^*−/−*^ double mutants compared to *Lrp6* ^*−/−*^ mutants at E9.5, prior to the onset of neurogenesis.

**FIGURE 4:**
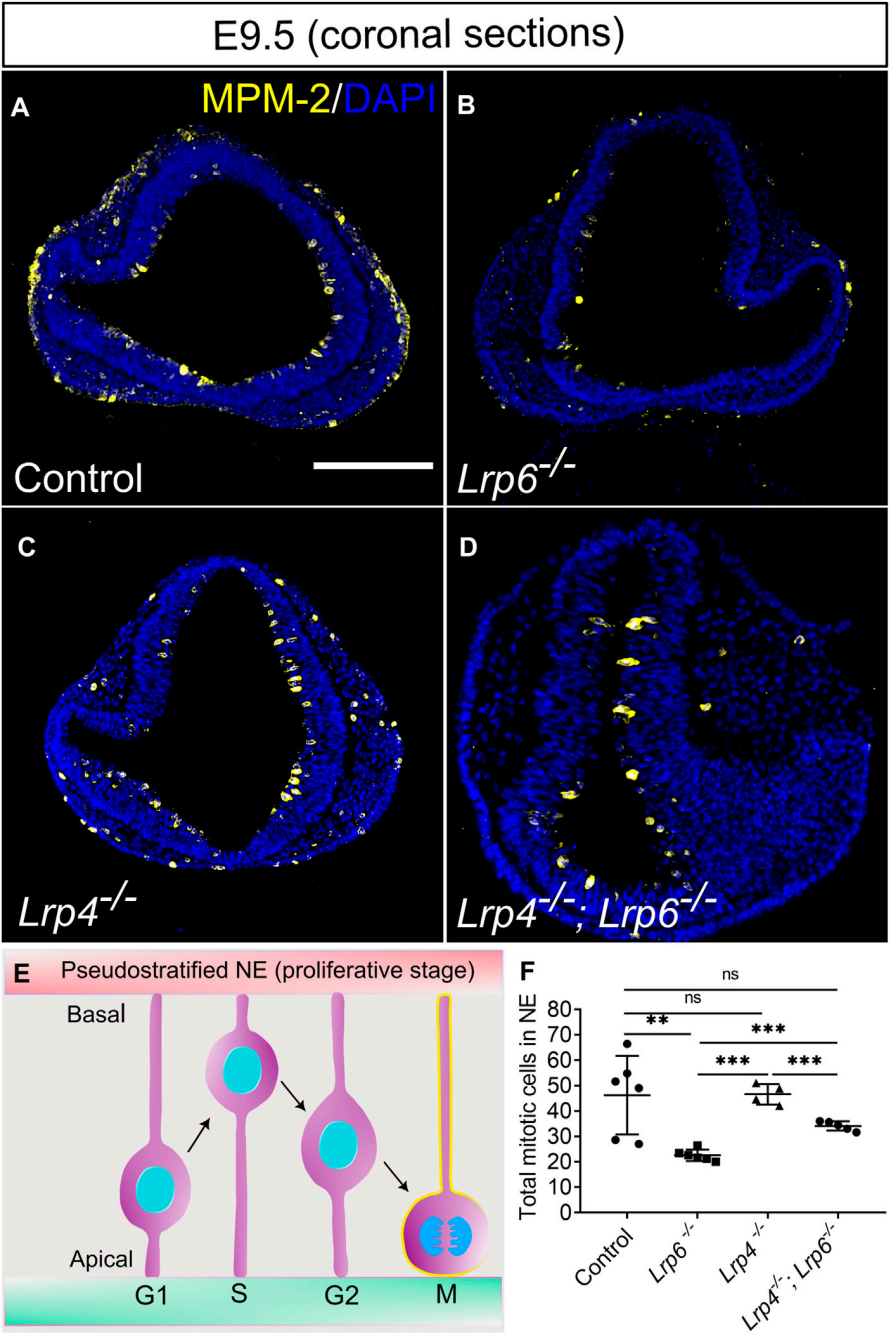
Decreased mitotic activity in LRP6-deficient neuroepithelium is rescued by ablation of LRP4. **(A–D)** Immunostaining for the mitosis marker MPM-2 (marks all cell in M-Phase) to visualize and quantify mitotic cells within the neuroepithelium on E 9.5 coronal forebrain sections. MPM-2 positive cells were detected at the apical side of the neuroepithelium facing the ventricle [depicted in the schematic, **(E)**] in wild-type controls **(A)** and *Lrp4* ^*−/−*^ embryos **(C)** in similar numbers. *Lrp6* ^*−/−*^ forebrain neuroepithelium sections showed significantly lower numbers of mitotic cells **(B)** compared to wild-type controls and *Lrp4* ^*−/−*^ single mutants. *Lrp4* ^*−/−*^; *Lrp6* ^*−/−*^ double mutants **(D)** had similar numbers of mitotic neuroepithelial cells as wild-type controls and therefore showed a clear rescue of mitotic activity compared to *Lrp6* ^*−/−*^ single mutants. Scale bar: 200 μm. **(F)** To quantify mitotic cells within the neuroepithelium, neural progenitor cells that stained positive for mitosis marker MPM-2 were counted manually on six coronal sections of the forebrain for each embryo. Number of embryos for each genotype: controls *n* = 6, *Lrp6* ^*−/−*^ *n* = 6, *Lrp4* ^*−/−*^ *n* = 4; *Lrp4* ^*−/−*^; *Lrp6* ^*−/−*^ *n* = 5; The mean values of MPM-2 cell count for each section was calculated and correlated to DAPI counts. Statistics: one-way ANOVA; NE: neuroepithelium. NE: neuroepithelium.

At E9.5, *Lrp6* ^*−/−*^ single mutant embryos showed a notable decrease in the number of mitotic cells in the neuroepithelium compared to wild-type embryos (Figure 4). Immunofluorescence staining with the antibody MPM-2 (mitotic protein monoclonal 2) was used to visualize and quantify mitotic cells within the neuroepithelium. Interestingly, a significantly higher number of mitotic cells in the entire neuroepithelium of the forebrain was detected in *Lrp4* ^*−/−*^; *Lrp6* ^*−/−*^ double mutants compared to *Lrp6* ^*−/−*^ single mutants at E9.5. No significant difference in the count of mitotic cells within the neuroepithelium was observed in *Lrp4* ^*−/−*^ embryos compared to wild types (Figure 4). Thirty percent of all *Lrp4* ^*−/−*^; *Lrp6* ^*−/−*^ double mutants also developed sporadically excrescences in the neuroepithelium with areas of abnormally high numbers of mitotic cells and an aberrant cellular organization (Supplementary Figure S4A). Neuroepithelial cells in these excrescences were positive for SOX2 and therefore retained their progenitor character (Supplementary Figure S4B). To assess whether the increased number of mitotic cells in the entire neuroepithelium of *Lrp4* ^*−/−*^; *Lrp6* ^*−/−*^ double mutants compared to *Lrp6* ^*−/−*^ single mutants retain their neuronal progenitor character, we analysed the SOX2 expression pattern and quantified fluorescence intensity levels for SOX2 in samples of all genotypes including *Lrp4* ^*−/−*^; *Lrp6* ^*−/−*^ double mutants with and without excrescences. Overall SOX2 levels in the neuroepithelium were similar between all genotypes, only *Lrp4* ^*−/−*^ embryos showed slightly higher levels compared to *Lrp6* ^*−/−*^ mutants (Supplementary Figure S4C). Together, these results suggest that the decreased number of mitotic neuronal progenitors in *Lrp6* ^*−/−*^ mutants at E9.5 is rescued in *Lrp4* ^*−/−*^; *Lrp6* ^*−/−*^ double mutants and that increased numbers of mitotic cells are not associated with a change in the fate of the neuroepithelial cells in double mutants as indicated by a normal SOX2 pattern.

### 3.5 LRP4 function modulates LRP6-dependent mitotic activity in hTERT-RPE1 cells

**FIGURE 5:**
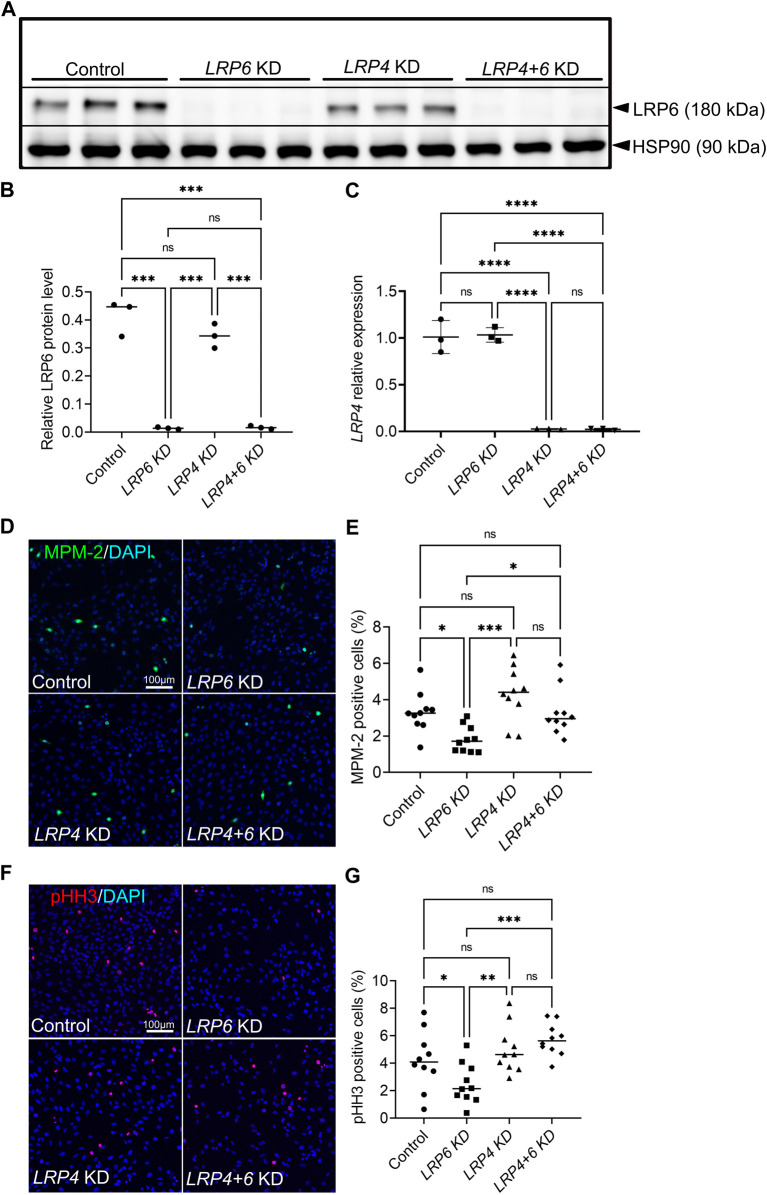
LRP4 modulates of LRP6-dependent mitotic activity in human TERT RPE-1 cells. **(A, B)** Western blot analysis and quantification of LRP6 protein levels after *Lrp6* siRNA and control siRNA treatment respectively, demonstrated significantly lower LRP6 protein levels in hTERT RPE-1 cells after LRP6 silencing and simultaneous LRP4 and LRP6 silencing compared to controls and compared to LRP4 silencing only. These results validate the LRP6 silencing and demonstrate that there is no up- or downregulation of LRP4 levels in *Lrp6* siRNA treated cells. Quantification of LRP6 levels was normalized to HSP90 (heat shock protein 90) signals. Three independent experiments in triplicates (technical replicates) are summarized in the graph. Significance assessed by one-way ANOVA. **** *p* ≤ 0.0001; data are mean ± s.d. **(C)** *LRP4* siRNA mediated silencing was validated by quantitative RT-RCR. Significantly lower *LRP4* transcription was detected in hTERT RPE-1 cells after LRP4 silencing and simultaneous LRP4 and LRP6 silencing compared to controls and compared to LRP6 silencing only. The results demonstrate that there is no up- or downregulation of *LRP6* mRNA levels in *LRP4* siRNA treated cells. Three independent experiments in triplicates are summarized in the graph. Significance assessed by one-way ANOVA. *p* value: **** *p* ≤ 0.0001; data are mean ± s.d. **(D–E)** MPM-2 positive cells are detected in hTERT RPE-1 cultures by immunocytochemistry. MPM-2 stains the cytoplasm from early prophase through metaphase, anaphase and telophase1. LRP4 silencing resulted in similar numbers of mitotic cells compared to controls. Significantly less mitotic cells were counted in cultures treated with siRNA to silence LRP6 compared to control siRNA treated cultures or *LRP4* siRNA treated cultures. This decrease caused by LRP6-depletion was rescued by simultaneous silencing of LRP4 and LRP6. Scale bar: 100 μm. **(E)** MPM-2 positive cell number quantification was normalized to DAPI positive cells. 10 images of 581 μm^2^ were measured in each well. Quantification of three independent experiments with triplicates are summarized in the graph. Significance assessed by one-way ANOVA. *p* values: * *p* < 0.05, ***p ≤ 0.001; data are mean ± s.d. **(F–G)** Detection of phospho-histone H3 (pHH3) by immunocytochemistry in the nuclei of cells during M-phase. Similar to the results obtained by quantification of MPM-2 positive cells, significantly less mitotic cells were counted in cultures treated with siRNA to silence LRP6 compared to control or *LRP4* siRNA treated cultures. This decrease caused by LRP6-depletion was rescued by simultaneous silencing of LRP4 and LRP6. Scale bar: 100 μm. **(G)** pHH3 positive cell number quantification was normalized to DAPI positive cells. 10 images of 581 μm^2^ were measured in each well. Quantification of three independent experiments with triplicates are summarized in the graph. Significance assessed by one-way ANOVA. *p* values: * *p* < 0.05, ** *p* ≤ 0.01, ***p ≤ 0.001; data are mean ± s.d.; KD: siRNA mediated knockdown (silencing).

To test whether the function of LRP4 in balancing mitotic activity is context-dependent and restricted to the developing forebrain or whether this function can be seen in a more general context, we used human retinal pigment epithelial (hTERT RPE-1) cells. Silencing of LRP4 and LRP6, respectively or of both receptors simultaneously, recapitulated results from the different genotypes we tested in the mouse model. Efficient knockdown using siRNA was confirmed by decreased levels of *LRP4* and *LRP6/*LRP6, respectively (Figures 5A–C). Consistent with our results in *Lrp4* ^*−/−*^ and *Lrp6* ^*−/−*^ single mutants and *Lrp4* ^*−/−*^; *Lrp6* ^*−/−*^ double mouse mutants, we detected significantly lower numbers of mitotic cells in human TERT RPE-1 cell cultures treated with *LRP6* siRNA compared to cultures treated with control siRNA, while silencing both *LRP4* and *LRP6* resulted in significantly higher levels of cells positive for MPM-2 (Figures 5D, E) and of cells positive for the other mitosis marker phospho-histone 3 (pHH3) (Figures 5F, G), respectively, compared to the *LRP6* single knockdown. Mitotic cell levels in cultures with simultaneous knockdown of LRP4 and LRP6 were similar to control levels, indicating that LRP5 or other factors may compensate for the loss of LRP6 in the absence of LRP4.

### 3.6 Genetic inactivation of LRP4 rescues impaired canonical WNT activity in *Lrp6* ^*−/−*^ mutants

**FIGURE 6:**
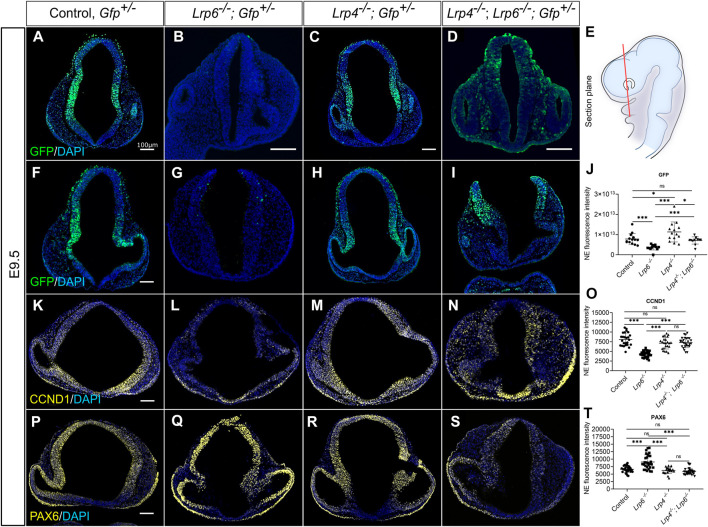
Genetic inactivation of LRP4 rescues impaired canonical WNT activity and downstream target gene expression in *Lrp6* ^*−/−*^ mutants. **(A–J)** *TCF/Lef:H2B-GFP* transgenic mouse line was used to visualize and quantify WNT/ß-catenin-signalling in neuroepithelial cells of all *Lrp* genotypes and controls. Quantification of GFP immunohistochemistry signals were performed on coronal forebrain sections from E9.5 mouse embryos. **(E)** Section plane for representative images is indicated in the schematic. **(A–D)** Representative images show forebrain sections from E9.5 embryos with closed cranial neural tube for all genotypes. **(F–I)** Images show forebrain sections from E9.5 embryos with open cranial neural tube phenotype for *Lrp6* ^*−/−*^; *Gfp* ^*+*^ mutants and *Lrp4* ^*−/−*^; *Lrp6* ^*−/−*^; *Gfp* ^*+*^ double mutants. Wild-type; *Gfp* ^*+*^ controls and *Lrp4* ^*−/−*^; *Gfp* ^*+*^ embryos never showed neural tube defects. Immunohistochemistry images in **(A, F)** show the pattern and intensity of GFP signals in wild-type controls, which displayed WNT/ß-catenin activity in the dorsolateral domain of the forebrain neuroepithelium. In age-matched and plane-matched forebrain sections of *Lrp4* ^*−/−*^; *Gfp* ^*+*^ embryos **(C, H)**, a similar pattern and intensity as in wild-type; *Gfp* ^*+*^ controls were observed. In the neuroepithelium of *Lrp6* ^*−/−*^; *Gfp* ^*+*^ forebrains **(B, G)** significantly less GFP signal intensity was detected compared to controls, indicating a decrease in canonical WNT signalling activity. *Lrp4* ^*−/−*^; *Lrp6* ^*−/−*^; *Gfp* ^*+*^ double mutants **(D, I)** showed GFP intensity levels that were significantly higher than in *Lrp6* ^*−/−*^; *Gfp* ^*+*^ single mutants and similar to wild-type; *Gfp* ^*+*^ controls, suggesting a clear rescue of canonical WNT signalling activity upon depletion of LRP4 function in *Lrp6* ^*−/−*^ mutants. The reduced WNT/ß-catenin activity in *Lrp6* ^*−/−*^; *Gfp* ^*+*^ single mutants and the rescue of canonical WNT/ß-catenin activity in LRP6-deficient forebrains by genetic ablation of LRP4 was also observed regardless of whether the embryos had a closed neural tube **(B, D)** or displayed cranial NTDs **(G, I)**. **(J)** Graph shows quantification of mean GFP signal fluorescence intensity, *y* axis: fluorescence intensity in the entire neuroepithelium (NE). A total of 4 - 6 coronal sections from each embryo were examined from E9.5 wild-type; *Gfp* ^*+*^ embryos (*n* = 3), *Lrp6* ^*−/−*^; *Gfp* ^*+*^ mutants (*n* = 3), *Lrp4* ^*−/−*^; *Gfp* ^*+*^ mutants (*n* = 3) and *Lrp4* ^*−/−*^; *Lrp6* ^*−/−*^; *Gfp* ^*+*^ double mutants (*n* = 3). Scatter plot presents mean ± s.d.; the significance was assessed with student t-test; *p* values: * *p* < 0.05, *** *p* < 0.001. NE: neuroepithelium. **(K–N)** Cyclin D1 (CCND1) is a known WNT/ß-catenin downstream target gene and a cell cycle regulator. Images show immunohistochemistry for Cyclin D1 on coronal forebrain sections. *Lrp4* ^*−/−*^; *Lrp6* ^*−/−*^ double mutants exhibited significantly stronger signals for Cyclin D1 in the neuroepithelium compared to *Lrp6* ^*−/−*^ single mutants, which had dramatically reduced Cyclin D1 levels compared to controls. *Lrp4* ^*−/−*^ single mutants displayed similar levels of Cyclin D1 as wild-type controls. **(O)** Graph shows quantification of Cyclin D1 immunohistochemistry signal intensity, *y* axis: mean fluorescence intensity in the entire neuroepithelium. Immunofluorescence intensity of Cyclin D1 measured in the neuroepithelium from E9.5 wild-type; *Gfp* ^*+*^ embryos (*n* = 3), *Lrp6* ^*−/−*^; *Gfp* ^*+*^ mutants (*n* = 4), *Lrp4* ^*−/−*^; *Gfp* ^*+*^ mutants (*n* = 3) and *Lrp4* ^*−/−*^; *Lrp6* ^*−/−*^; *Gfp* ^*+*^ double mutants (*n* = 3). A total of 7–12 coronal sections from each embryo were examined. Scatter plot presents mean ± s.d.; the significance was assessed with one-way ANOVA; *p* value: *** *p* < 0.001. **(P–S)** PAX6 is another downstream target of the WNT/ß-catenin pathway but in contrast to Cyclin D1 negatively regulated. Accordingly, *Lrp6* ^*−/−*^ embryos showed stronger signals for PAX6 compared to wild types and compared to *Lrp4* ^*−/−*^ mutants, which showed a normal pattern for PAX6 in the mediolateral anterior neural tube. *Lrp4^−/−^; Lrp6^−/−^* mutants showed PAX6 signal intensity and PAX6 pattern comparable to wild-type controls and therefore a rescue of impaired PAX6 protein levels in Lrp6^−/−^ single mutants. **(T)** Graph shows quantification of PAX6 immunohistochemistry signal intensity, *y* axis: mean fluorescence intensity in the entire neuroepithelium. Immunofluorescence intensity of GFP measured in the neuroepithelium from E9.5 wild-type; *Gfp* ^*+*^ embryos (n = 3), *Lrp6* ^*−/−*^; *Gfp* ^*+*^ mutants (*n* = 4), *Lrp4* ^*−/−*^; *Gfp* ^*+*^ mutants (*n* = 3) and *Lrp4* ^*−/−*^; *Lrp6* ^*−/−*^; *Gfp* ^*+*^ double mutants (*n* = 3). A total of –15 coronal sections from each embryo were examined. Scatter plot presents mean ± s.d.; the significance was assessed with one-way ANOVA; *p* value: *** *p* < 0.001. Scale bars: 100 µm.

To investigate if the rescue of mitotic neuronal precursor abundance is associated with augmented canonical WNT signalling activity, we crossed the *Lrp4* ^*+/−*^; *Lrp6* ^*+/−*^ double heterozygous mice onto the *TCF/Lef:H2B-GFP* transgenic WNT signalling reporter mice. The expression of the H2B-EGFP fusion protein under the control of six copies of a T cell specific transcription factor/lymphoid enhancer-binding factor 1 (*Lef1*) response element and a heat shock protein 1B minimal promoter enabled us to visualize and quantify WNT/β-catenin signalling activity during early forebrain development. In this study, *Lrp4*, *Lrp6*, and *Lrp4*; *Lrp6* double mutant or control mice carrying one allele of the TCF/Lef:H2B-GFP reporter are referred to as *Gfp* ^*+/−*^ (e.g., *Lrp4* ^*−/−*^; *Gfp* ^*+/−*^). GFP signals, indicating WNT-responsive neuronal progenitors in the dorsal and lateral neural tube of *Lrp4* ^*−/−*^; *Gfp* ^*+/−*^ embryos, were increased compared to wild-type controls at E9.5 (Figures 6A–J), suggesting slightly elevated canonical WNT activity upon loss of LRP4 in the early forebrain. In contrast, the forebrain neuroepithelium of *Lrp6* ^*−/−*^; *Gfp* ^*+/−*^ embryos showed significantly lower WNT reporter levels than wild-type controls, regardless of whether the neural tube was open or closed (Figures 6A–J). Only few GFP-positive cells were observed within the dorsolateral forebrain neuroepithelium in *Lrp6* ^*−/−*^ mutants with open or closed anterior neural tube at E9.5 (Figures 6B, G). *Lrp4* ^*−/−*^; *Lrp6* ^*−/−*^ double mutant embryos exhibited significantly increased GFP signals in the forebrain neuroepithelium at E9.5 compared to *Lrp6* ^*−/−*^ single mutants, indicating a rescue of canonical WNT activity in *Lrp6* ^*−/−*^ mutants upon loss of LRP4. Of note, the elevated WNT responsive reporter activity in *Lrp4* ^*−/−*^; *Lrp6* ^*−/−*^ double mutants was observed regardless of whether the anterior neural tube was closed or still open as part of the phenotype at E9.5 (Figures 6D, I). Our findings on the TCF/Lef:H2B-GFP reporter activity in *Lrp6* ^*−/−*^ embryos and *Lrp4* ^*−/−*^; *Lrp6* ^*−/−*^ double mutant embryos confirmed that *Lrp6* ^*−/−*^ single mutants have reduced WNT target gene activation in the forebrain compared to wild types. Importantly, depletion of LRP4 in *Lrp6* ^*−/−*^ mutants could rescue decreased WNT activity that ß-catenin-dependent gene transcription in embryos with closed and open neural tube at E9.5, suggesting that LRP4 activity can significantly influence canonical WNT signalling in the forebrain neuroepithelium and thereby mitotic activity/proliferation of neuronal precursors, but not the morphogenesis of neural tube closure. To support the results on canonical WNT pathway activity obtained by quantifying the TCF/Lef:H2B-GFP reporter activity, we analysed mRNA expression of the endogenous transcription factor, lymphoid enhancing factor (*Lef1*). Whereas it has long been recognized that WNT induces signalling through the TCF/LEF1 cascade (Behrens et al., 1996), it recently became clear that WNT3a can also directly induce transcription of *Lef1* (Hovanes et al., 2001; Filali et al., 2002; Driskell et al., 2004; Nguyen et al., 2009). Therefore, LEF1 is a mediator and target of WNT/ß-catenin signalling. *Lef1* expression was markedly reduced in *Lrp6* ^*−/−*^ single mutant embryos. The *Lef1* expression pattern in *Lrp4* ^*−/−*^ embryos showed partially stronger signals compared to wild types, in particular in the dorsal midline of the anterior neural tube, a domain that showed few transcripts in wild types (Supplementary Figure S5C). In the dorso-lateral forebrain domain of *Lrp4* ^*−/−*^; *Lrp6* ^*−/−*^ double mutant embryos, *in situ* hybridization for *Lef1* showed higher levels compared to the neural tube in *Lrp6* ^*−/−*^ embryos (Supplementary Figures S5G, H).

Next, we aimed to investigate whether increased WNT reporter activity and higher endogenous *Lef1* levels in *Lrp6* ^*−/−*^; *Lrp4* ^*−/−*^ double mutants could rescue the expression of ß-catenin/LEF1 target genes besides the induction of *Lef1* itself. We quantified Cyclin D1 protein levels as it is a known downstream target of the canonical WNT pathway and as Cyclin D1 activity is required for cell cycle G1/S transition (Baldin et al., 1993; Shtutman et al., 1999; Lecarpentier et al., 2019). The results showed that *Lrp6* ^*−/−*^, *Lrp4* ^*−/−*^ double mutants exhibited significantly stronger signals for Cyclin D1 in the forebrain compared to *Lrp6* ^*−/−*^ single mutants, which had dramatically reduced Cyclin D1 levels compared to controls (Figures 6K–O). *Lrp4* ^*−/−*^ single mutants displayed similar levels of Cyclin D1 as wild-type controls (Figure 6O), suggesting that slightly elevated WNT activity levels in *Lrp4* ^*−/−*^ mutants were not sufficient to increase the average Cyclin D1 levels in the forebrain neuroepithelium. We also explored a potential functional link between the WNT pathway and PAX6 in the developing brain (Assimacopoulos et al., 2003; Gray et al., 2013). Consistent with the results from other labs (Zhou et al., 2006; Gray et al., 2013), *Lrp6* ^*−/−*^ embryos showed strongly enhanced signals for PAX6, mainly in the dorsal midline of the forebrain at E9.5 compared to wild types and compared to *Lrp4* ^*−/−*^ mutants, which showed a normal pattern and expression level for PAX6 in the mediolateral anterior neural tube (Figures 6P–T). These data suggest that decreased WNT/ß-catenin activity directly or indirectly leads to an expansion of the PAX6 protein expression domain and an increase in PAX6 levels. Interestingly, loss of LRP4 in *Lrp6* ^*−/−*^ mutants rescued the abnormal PAX6 pattern and levels in the forebrain (Figures 6S, T).

### 3.7 LRP4 is a modulator of LRP6-dependent Cyclin D1 expression in human TERT RPE-1 cells

**FIGURE 7:**
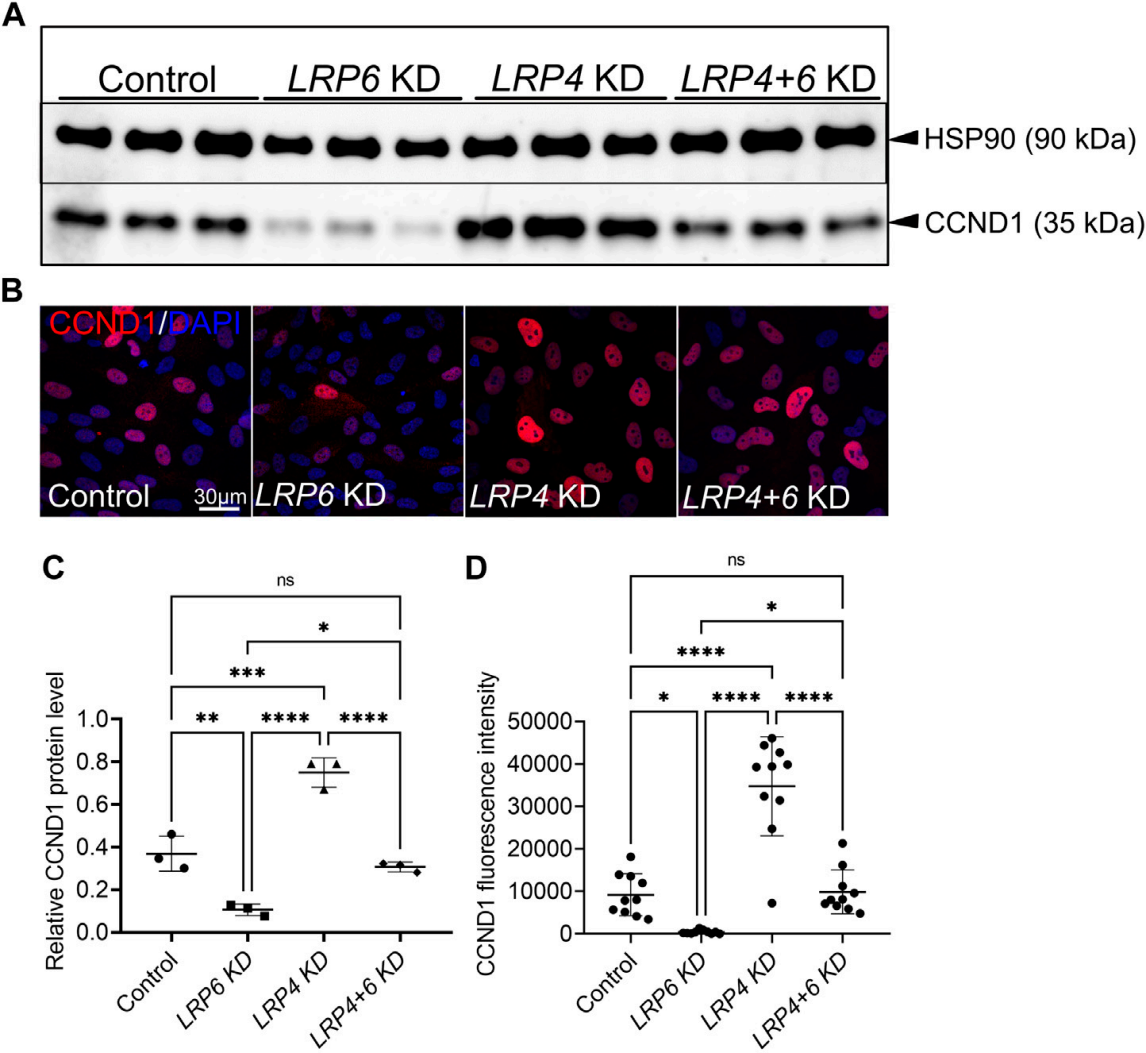
LRP4 modulates of LRP6-dependent Cyclin D1 levels in human TERT RPE-1 cells. **(A, C)** Western blot analysis and quantification of Cyclin D1 (CCND1) protein levels after *Lrp6* siRNA and control siRNA treatment respectively, demonstrated significantly lower Cyclin D1 protein levels hTERT RPE-1 cells after LRP6 silencing. Significant upregulation of Cyclin D1 levels were observed in cultures where LRP4 was silenced compared to cultures treated with control siRNA. Simultaneous silencing of LRP4 and LRP6 showed Cyclin D1 levels comparable with controls and therefore a rescue of decreased Cylin D1 levels in cells with LRP6 silencing. Quantification of Cyclin D1 levels was normalized to HSP90 (heat shock protein 90) signals. Three independent experiments in triplicates are summarized in the graphs. Significance assessed by one-way ANOVA. *p* values: * *p* < 0.05, ** *p* ≤ 0.01, ***p ≤ 0.001, **** *p* ≤ 0.0001; data are mean ± s.d. **(B, D)** Cyclin D1 positive cells are detected in hTERT RPE-1 cultures by immunocytochemistry. LRP4 silencing resulted in higher levels of Cyclin D1 positive cells compared to controls. Significantly less Cyclin D1 positive cells were counted in cultures treated with siRNA to silence LRP6 compared to control siRNA treated cultures. This decrease in Cyclin D1 levels, caused by LRP6-depletion, was rescued by simultaneous silencing of LRP4 and LRP6. Scale bar = 30 μm. Cyclin D1 signal intensity quantification was normalized to DAPI positive cell counts. 10 images of 160 μm^2^ were measured in each well. Quantification of three independent experiments with triplicates are summarized in the graph. Significance assessed by one-way ANOVA. *p* values: * *p* < 0.05, **** *p* ≤ 0.0001; data are mean ± s.d.; KD: siRNA mediated knockdown (silencing).

Since aberrant WNT and proliferative activities not only cause congenital defects, but also degenerative diseases and cancers (Nusse and Clevers, 2017), we tested whether the function of LRP4 as a modulator of WNT pathway activation and consequently of Cyclin D1 levels is restricted to the murine forebrain or context-independent. Silencing of LRP6 in human TERT RPE-1 cells caused a clear decrease in levels of the WNT pathway downstream target Cyclin D1 as observed by Western blot analyses and immunocytochemistry, which was rescued by simultaneous silencing of LRP4 and LRP6 (Figure 7). Significant upregulation of Cyclin D1 levels were observed in cultures with LRP4 silencing compared to cultures treated with control siRNA. Of note, while no significant upregulation of Cyclin D1 in LRP4-deficient murine forebrain neuroepithelium was observed despite elevated WNT activity (Figure 6O), loss of LRP4 in human RPE cells resulted in elevated Cyclin D1 levels. The observed difference in Cyclin D1 upregulation between *in vivo* and *in vitro* models suggests that different WNT activity thresholds are needed to increase Cyclin D1 levels.

Altogether, data on WNT reporter activity and analysis of WNT downstream targets in double *Lrp6* ^*−/−*^; *Lrp4* ^*−/−*^ mouse mutants suggest that loss of LRP4 on an *Lrp6* ^*−/−*^ background can ameliorate impaired canonical WNT/ß-catenin/LEF pathway activation in the early murine forebrain, supporting the hypothesis that LRP4 counteracts LRP6-mediated canonical WNT signalling and that *Lrp4* is a genetic modifier for phenotype caused by loss of LRP6. Further, gene silencing experiments in a human cell culture line could recapitulate the functional interaction of LRP4 and LRP6 in regulating mitotic activity. The findings suggest that LRP4 plays an essential function in balancing WNT/ß-catenin signalling activity not only in the murine forebrain but also in human cells. The study’s results provide insight into the essential role of LRP4 in regulating mitotic activity and suggest that targeting LRP4 may be a potential therapeutic approach for conditions related to aberrant canonical WNT and proliferative activities.

## 4 Discussion

The first WNT protein was discovered by Nusse and Varmus 4 decades ago years ago (Nusse and Varmus, 1982), and since then our understanding of WNT signalling in the developing brain has steadily increased. It is therefore not surprising that WNT signalling also plays a pivotal role during formation of the mammalian forebrain, a highly complex developmental process (Harrison-Uy and Pleasure, 2012). Here we focused on the early stages of forebrain development prior to neurogenesis. At these embryonic stages the neural plate is composed of a single layer of cells, the neuroepithelial cells, which form the neuroepithelium. The neuroepithelium looks layered (pseudostratified), because the nuclei of neuroepithelial cells migrate up and down the apical–basal axis during the cell cycle (interkinetic nuclear migration). Highly regulated proliferative activity of the neuroepithelial cells, the neuronal precursor cells (NPCs) as well as coordinated patterning and morphogenetic processes are crucial for neural fold formation, elevation, and finally closure of the neural tube around mid-gestation. The WNT pathway plays a major role in proliferation and morphogenesis during forebrain formation and perturbations in canonical and non-canonical WNT signalling lead to defects in neural tube closure (Freese et al., 2010; Engelhardt et al., 2022). The exceptional sensitivity of the forebrain and associated craniofacial structures to WNT activity highlights the fact that formation of the embryonic brain and head strongly depends on fine-tuned regulation of the localization and level of WNT signalling activity in the progenitor tissues (Lagutin et al., 2003; Lewis et al., 2007; 2008; Fossat et al., 2011). Crucial for proper WNT signal transduction is the extracellular docking site of WNT proteins, which comprises the transmembrane receptors Frizzled (FZD) and LRP5/6 (Robb and Tam, 2004; MacDonald and He, 2012). While the role of FZD receptors during brain development is well documented (Burns et al., 2008; Wang et al., 2016), less is known about the involvement of LRP5 and LRP6 in WNT-related mammalian early forebrain formation (Zhou et al., 2006; Gray et al., 2010; Gray et al., 2013; Fossat et al., 2011).

Our study provides evidence that besides LRP5/6 also LRP4, another member of the LDL-receptor protein family, plays an important role in WNT signal transduction during early forebrain development. While the concept of WNT binding to the FZD-LRP receptor complex and leading to activation of WNT downstream targets is well understood, less is known about how this ligand-receptor interaction can be modulated to alter WNT signalling outcomes (MacDonald and He, 2012). However, there is growing evidence that modulation of the FZD co-receptor complex plays an important role in regulating WNT target gene expression. For example, LRP4 can modulate the function of LRP5/6 in conjunction with the WNT-inhibiting protein WISE during the development of non-neuronal tissue (Ohazama et al., 2008; Ahn et al., 2013; Ahn et al., 2017; Kawasaki et al., 2018). Our study provides genetic evidence that modulation of LRP5/6 function by LRP4 is also crucial for WNT target gene expression during mouse forebrain development.

### 4.1 Functional interaction between LRP4 and LRP5

LRP4 and LRP5 are important in various physiological processes, including bone metabolism, cardiovascular function, and neuronal development at later embryonic stages (Ciani and Salinas, 2005; Salinas and Zou, 2008; Huang et al., 2016). Functional interaction of LRP4 and LRP5 has been suggested in bone formation (Leupin et al., 2011; Choi and Robling, 2021). However, a potential interaction in forebrain development remained obscure. During early forebrain patterning and morphogenesis, *Lrp4* ^*−/−*^ mutants show no obvious defects, suggesting that LRP4 plays a limited role in early forebrain development. Similarly, *Lrp5* ^*−/−*^ mutants develop a normal forebrain. These results indicate that LRP4 and LRP5 are dispensable for the intact gross morphology of the early forebrain and that LRP6 likely plays a more important role in the early morphogenetic processes of forebrain development. However, when LRP4 and LRP5 are both deleted, double mutants exhibit a cranial neural tube defect (NTD), suggesting that these two genes may act together in a complementary or compensatory manner to regulate neural tube closure. The experiment also demonstrates that LRP6 alone, in absence of LRP4 and LRP5, seems to be insufficient to regulate canonical and/or non-canonical WNT signalling to drive proper neural tube morphogenesis, particularly closure of the anterior neural pore. Furthermore, the early lethality of *Lrp4* ^*−/−*^; *Lrp5* ^*−/−*^ double mutants around mid-gestation highlights the complexity of gene function and the interplay between different genes in regulating developmental processes.

### 4.2 Genetic ablation of *Lrp4* partially rescues neuroepithelial hypoplasia phenotypes in *Lrp6* ^*−/−*^ mutants but not neural tube closure defects

Previous gene targeting experiments have revealed that LRP5 and LRP6 play distinct roles in development (Ren et al., 2021), but also exhibit functional redundancy, as double null mutants show early embryonic lethality before mid-gestation (Kelly et al., 2004), while *Lrp5* ^*−/−*^ single mutants are viable and *Lrp6* ^*−/−*^ mice survive until birth. To further investigate the functional interaction of LRP family members in WNT signalling, we next analysed *Lrp4* ^*−/−*^; *Lrp6* ^*−/−*^ double mutants.

Our study showed that *Lrp6* ^*−/−*^ single mutants exhibit forebrain hypoplasia and a decreased number of mitotic neuronal precursors at E9.5, consistent with previous studies (Gray et al., 2010; Gray et al., 2013). However, whereas Gray and colleagues as well as our lab observed a reduced number of cell divisions in *Lrp6* ^*−/−*^ embryos already at E9.5, Zhou and colleagues observed significant hypoplasia in *Lrp6* ^*−/−*^ mutants only at later embryonic stages (Zhou et al., 2006). Consistent throughout all studies on *Lrp6* mutants, proliferation was impaired, whereas cell death did not increase. Our suggested role of LRP6 in maintaining proliferative capacity of neuronal precursors is in line with previous studies showing that WNT signalling regulates proliferation of cortical and hippocampal progenitor cells (Galceran et al., 2000; Lee et al., 2000; Chenn and Walsh, 2002). Previous studies have reported a pro-proliferative role of LRP6 also in non-neuronal cell types, i.e., vascular smooth muscle cell (Wang et al., 2004), breast cancer cells (Liu et al., 2010), and hepatocellular carcinoma cells (Tung et al., 2012).

We found that genetic ablation of *Lrp4* partially rescued impaired neuroepithelial cell proliferation and forebrain hypoplasia in *Lrp6* ^*−/−*^ mutants, indicating that loss of LRP4 function may counteract the negative effects of LRP6 deficiency on forebrain development and that, in this context, LRP5 can partially compensate for LRP6 function. The beneficial effect of LRP4 ablation on the proliferation in neuroepithelial cells, deficient for LRP6, was not restricted to mice but was also observed in human TERT RPE-1 cells. In light of these findings, we propose that the functional interaction between LRP4 and LRP6 may have relevance beyond forebrain development and may play a role in balancing cell proliferation and differentiation in various cell and tissue types in health and disease.

Another prominent phenotype in mice carrying either gain-of-function or loss-of-function mutations of *Lrp6* is neural tube defects (NTDs) with full penetrance of spina bifida and incomplete penetrance of anterior neural tube closure defects causing exencephaly (Pinson et al., 2000; Carter et al., 2005; Bryja et al., 2009; Gray et al., 2010; Gray et al., 2013; Zhou et al., 2010; Zhao et al., 2022). Multiple studies have reported patients with NTDs carrying mutations in the *LRP6* gene (Allache et al., 2014; Lei et al., 2015; Shi et al., 2018). Here, we observed anterior NTD in *Lrp6* ^*−/−*^ mutants with a penetrance of 32%, consistent with previous reports. In *Lrp4* ^*−/−*^; *Lrp6* ^*−/−*^ double mutant embryos we observed anterior (cranial) NTDs at the same frequency as in *Lrp6* ^*−/−*^ single mutants and therefore no rescue of cranial NTDs. Altogether these results suggest that *Lrp4* is a genetic modifier for proliferation defects, but not for anterior NTDs in *Lrp6* ^*−/−*^ mutants. We conclude that the interaction of LRP4 with LRP6 affects proliferation through the canonical WNT signalling pathway, but not neural tube closure processes regulated by non-canonical WNT/PCP signalling.

### 4.3 Genetic inactivation of LRP4 function can rescue impaired canonical WNT signalling activity in *Lrp6* ^*−/−*^ mutants

The lack of LRP4 and LRP6 in double mutants led to a significant increase in GFP reporter signals from the TCF/Lef reporter cassette in the forebrain neuroepithelium, regardless of whether they displayed cranial NTDs or had a closed neural tube, compared to single mutants lacking only LRP6 (Figure 6). These findings support the idea that impaired non-canonical WNT/PCP signalling is the primary underlying cause of the cranial NTDs in *Lrp6* ^*−/−*^ mutants, which is consistent with other studies (Bryja et al., 2009; Gray et al., 2013). Furthermore, it appears that LRP4 cannot efficiently modulate this pathway during early neural fold elevation. We detected *Lrp4* expression only after neural tube closure, and it is possible that LRP4 appears too late to be involved in the initial processes of neural tube closure or that *Lrp4* is present but expressed below detection levels during the relevant neurulation stages, and yet has no functional role in signalling pathways relevant to the initiation of neural tube closure. However, since *Lrp4* ^*−/−*^, *Lrp5* ^*−/−*^ double mutants displayed open anterior neuropore (ANP) with a penetrance of 88%, LRP4 in conjunction with LRP5 seems be involved in later stages of neural tube closure, such as final ANP closure at the 20 somite stage of development. Whether canonical and/or non-canonical WNT signalling or other signalling pathways are involved in this LRP4/5 influenced process remains to be investigated.

### 4.4 The functional link between canonical WNT/ß-catenin signalling and proliferation

The precise role of WNT signalling in balancing proliferation versus differentiation of neuronal progenitors is still not fully resolved (Munji et al., 2011; Da Silva et al., 2021). Previous studies have reported that canonical WNT signalling controls cell number by establishing a dorso-ventral mitogenic gradient (Dickinson et al., 1994; Ikeya et al., 1997; Megason and McMahon, 2002; Zechner et al., 2003). In line with the proliferation-stimulating effect of WNTs, we observed that dysregulated WNT/ß-catenin signalling in *Lrp6* ^*−/−*^ mutants resulted in altered expression levels of Cyclin D1 and PAX6, two important cell cycle regulators.

Cyclin D1 regulates progression from G1 to S phase of the cell cycle and higher levels of Cyclin D1 are associated with increased cell proliferation (Casimiro et al., 2012).

In *Lrp4* ^*−/−*^; *Lrp6* ^*−/−*^ double mutants, we observed a partial rescue of the reduced Cyclin D1 levels seen in *Lrp6* ^*−/−*^ single mutants (Figure 6), suggesting that LRP4 functions to counteract the pro-proliferative activity mediated by LRP6-dependent WNT/ß-catenin signalling in the early forebrain neuroepithelium prior to the onset of neurogenesis.

Furthermore, previous studies have established a functional link between the WNT/ß-catenin signalling pathway and *Pax6* expression in the developing brain (Assimacopoulos et al., 2003; Gray et al., 2013). Consistent with data from Gray and colleagues (Gray et al., 2013), we observed impaired mitotic activity and abnormally high levels of PAX6 protein in the neuroepithelium of *Lrp6* ^*−/−*^ mutants at E9.5, prior to the onset of neurogenesis. Additional loss of LRP4 in these *Lrp6* ^*−/−*^ single mutants led to increased canonical WNT/ß-catenin activity, which restored the abnormally high PAX6 levels and their reduced numbers of mitotic cells in the forebrain. Previous research suggests that PAX6 has a complex role in regulating proliferation and differentiation of neuronal progenitors, depending on the developmental stage and the tissue/cell types (Estivill-Torrus et al., 2002; Quinn et al., 2007; Hsieh and Yang, 2009; Quintana-Urzainqui et al., 2018). Based on the literature and our data we hypothesize that PAX6 is required for progenitor expansion after the onset of neurogenesis and that PAX6 gain-of-function in *Lrp6* ^*−/−*^ mutants can lead to suppression of mitotic activity in neuroepithelial cells at stages before E10.5.

### 4.5 Balancing WNT signalling requires functional interaction between LRP family members

**FIGURE 8:**
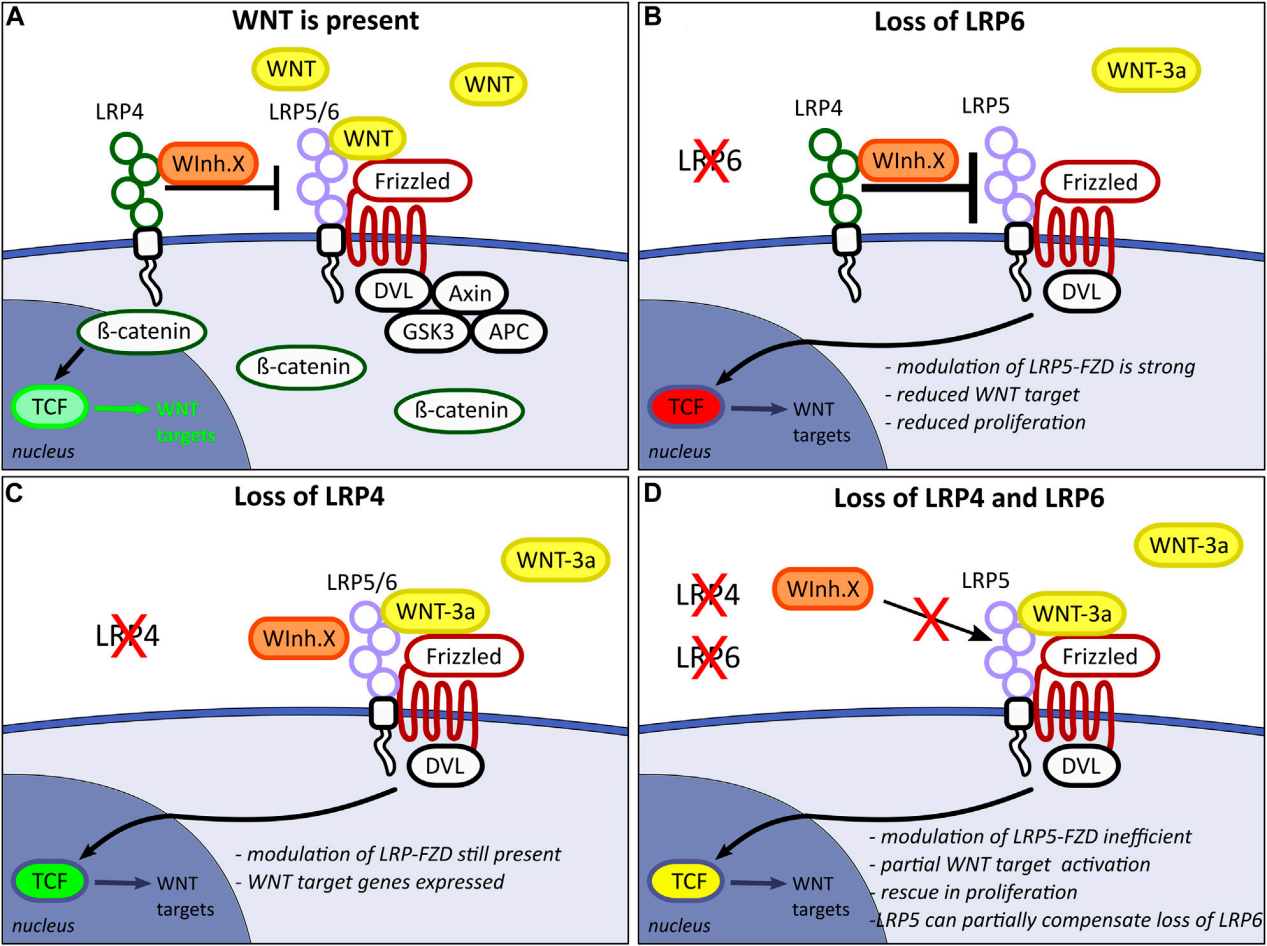
Hypothetical model for WNT pathway modulation by the LRP-Frizzled (FZD) complex in the developing forebrain. **(A)** LRP4 acts as an inhibitor on the LRP5/6-FZD complex and limits LRP5/6 binding capacity for WNT ligands, which consequently modulates WNT downstream target expression. LRP4 might present a WNT inhibitor X (WInh.X) to the LRP5/6-FZD complex. Model adapted from Ahn and colleagues (Ahn et al., 2013). **(B)** The loss of LRP6 function in the presence of WNT pathway inhibition by LRP4 leads to insufficient compensation by LRP5. This results in impaired binding of WNT3a to the LRP5-FZD complex, thereby inducing a significant reduction in the expression of WNT target genes. **(C)** In absence of LRP4, WNT inhibitor X can still bind to the LRP-FZD receptor complex. Slightly increased WNT/ß-catenin activity does not lead to significantly altered WNT downstream gene expression. **(D)** In *Lrp4* ^*−/−*^; *Lrp6* ^*−/−*^ double mutants, LRP4 can no longer present WNT inhibitor X to the LRP-FZD complex. Binding of WInh.X to LRP5 is too weak to have an inhibiting effect on the WNT/ß-catenin pathway activation. LRP5 can partially compensate for loss of LRP6 only in the absence of the WNT pathway inhibitor LRP4.

Based the results of this study, we conclude that LRP4 is a negative regulator of LRP6-mediated canonical WNT signalling, regulating mitotic activity of neuronal precursors in the early developing forebrain. Further, we conclude that LRP5 or an as-yet undetermined receptor can compensate for the loss of LRP6 as a FZD co-receptor in the absence of LRP4 (Figure 8). The notion that LRP4 and LRP6 have opposing effects on the WNT signalling pathway, with LRP4 suppressing and LRP6 promoting WNT signalling during forebrain development, is consistent with the studies by Ahn and colleagues, demonstrating that LRP4 acts as a negative regulator of WNT signalling, countering the positive effects of LRP6 during mammary gland and tooth development (Ahn et al., 2013; Ahn et al., 2017).

The rescue of forebrain hypoplasia in *Lrp4* ^*−/−*^; *Lrp6* ^*−/−*^ double mutants suggests that LRP6 alone is not fully sufficient to promote normal forebrain development, and the presence of LRP4 is necessary to balance the WNT pathway activity. Although LRP4 seems to be dispensable during early forebrain morphogenesis for intact gross anatomical forebrain structures, increased WNT activity observed in *Lrp4* ^*−/−*^ single mutants compared to wild types (Figure 6) might lead to aberrant specification of these early progenitors and ultimately possibly causing a range of neurodevelopmental defects and neurodegenerative disorders associated with LRP4 deficiency in later stage embryos and postnatally (DePew and Mosca, 2021). Further, although the slightly elevated WNT activity in LRP4 mutants was not sufficient to increase the average Cyclin D1 levels and mitotic activity in the forebrain neuroepithelium (Figures 4, 6), the observed sporadic locally appearing over-proliferative tumour-like neuroepithelial domains might be caused by increased canonical WNT signalling in LRP4 loss-of-function mutants (Supplementary Figures S4, S5).

Our data from the mouse models and human cell culture highlight the need for an intricate balance between WNT activation and inhibition not only in the ß-catenin activated downstream pathway but already at the level of WNT receptor complex function and modulation. Additionally, we suggest that the modulation of WNT signal transduction through receptor cross-talk is critical for maintaining the balance between proliferation and differentiation of neural progenitor cells in the embryonic forebrain.

Altogether, the study sheds light on the crucial roles of LRP4 and LRP6 in governing WNT signalling and forebrain development. The findings emphasize the importance of examining the interactions between various signalling pathways to comprehend the underlying pathophysiology of diseases. Further research is necessary to elucidate the precise mechanisms through which LRP4 and LRP6 modulate WNT signalling and forebrain development, with a particular focus on how LRP4 fine-tunes canonical WNT signalling to regulate proliferation.

## Data Availability

The original contributions presented in the study are included in the article/Supplementary Material, further inquiries can be directed to the corresponding authors.
