# Supplementary material for: Balancing WNT signalling in early forebrain development: The role of LRP4 as a modulator of LRP6 function
Source: Front Cell Dev Biol. 2023 Apr 7;11:1173688. doi: 10.3389/fcell.2023.1173688 (PMC10119419; doi:10.3389/fcell.2023.1173688)
Supplement: Supplementary file 1 [file DataSheet1.PDF]

SUPPLEMENTARY FIGURES AND FIGURE LEGENDS

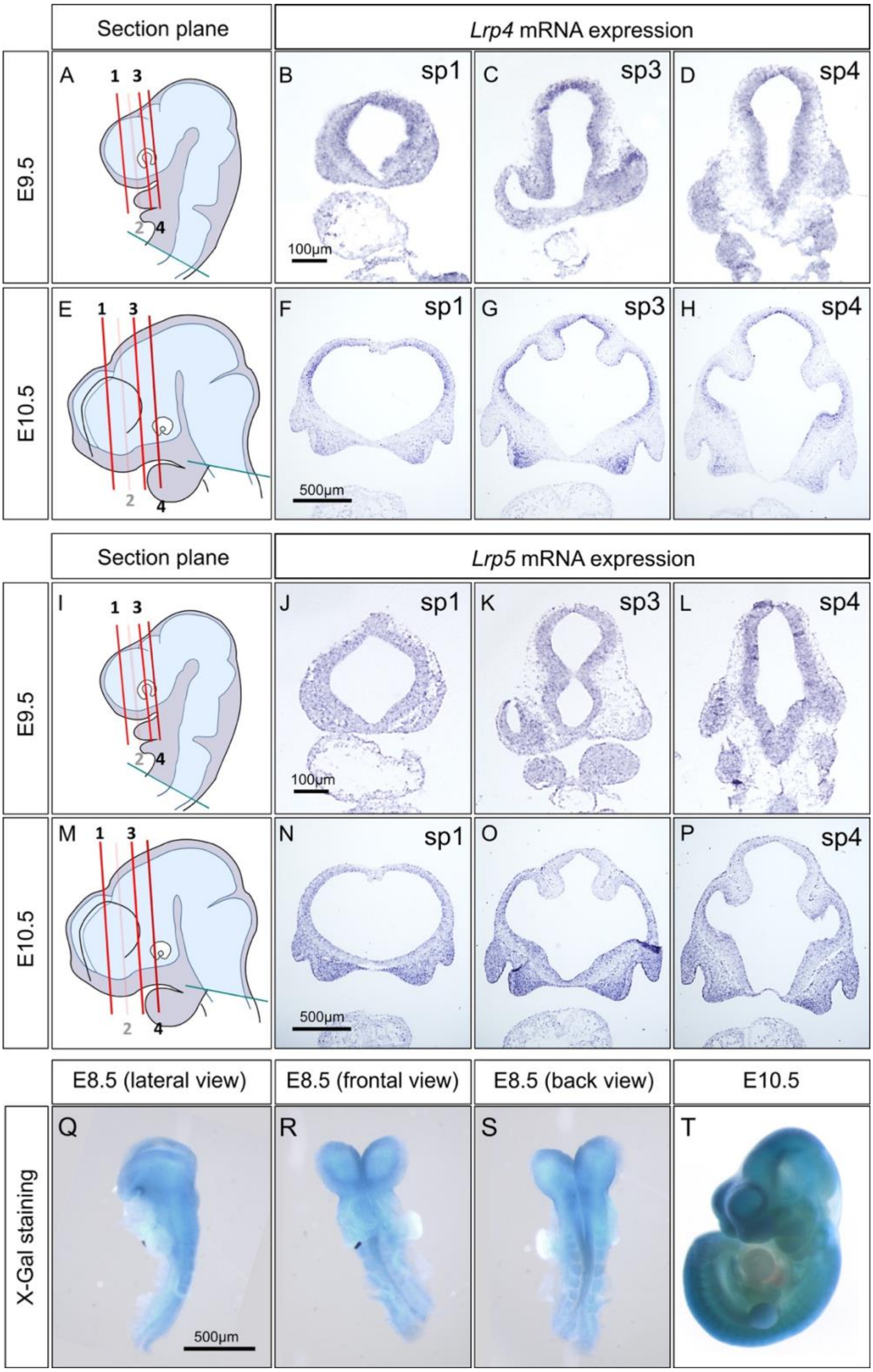

Supplementary Figure 1:

***Lrp4* and *Lrp5* show overlapping and distinct expression domains in the developing forebrain whereas *Lrp6* is ubiquitously expressed in neural tissue**

A - H: *Lrp4* expression pattern detected by *in situ* hybridization. **A**: Schematic indicates the coronal section planes (sp) at E 9.5, (results for section plane 2 are shown in Figure 1). **B - D**: *Lrp4* was expressed in the dorso-lateral domains of the neural tube; scalebar: 100 µm). **E:** Schematic indicates the coronal section planes at E 10.5, (results for section plane 2 are shown in Figure 1) **F - H**: *Lrp4* continued to be expressed in the dorso-lateral forebrain domains. The ventral midline is void of *Lrp4* transcripts, whereas the dorsal midline in section plane 3 and 4 showed strong *Lrp4* signals. Scalebar: 500 µm.

I - P: *Lrp5* expression pattern detected by *in situ* hybridization. **I**: Schematic indicates the coronal section planes at E 9.5, (results for section plane 2 are shown in Figure 1). **J - L:** *Lrp5* was expressed in the telencephalic and diencephalic forebrain at E 9.5; scalebar: 100 µm). **M:** Schematic indicates the coronal section planes at E 10.5, (results for section plane 2 are shown in Figure 1). **N - P**: At E10.5, *Lrp5* continued to be expressed in neuroepithelial cells with prominent signals in the ventral and lateral domains but little signal in the dorsal midline of all section planes. Scalebar: 500 µm.

Q - T: *Lrp6* expression pattern. Since the inserted gene trap vector of the Lrp6Gt(Ex187)Byg mouse line includes a ß-galactosidase reporter gene under control of the endogenous *Lrp6* promoter, gene expression of *Lrp6* was visualized by using X-Gal staining on Lrp6Gt(Ex187)Byg heterozygous embryos (*Lrp6*$^{+/-}$). *Lrp6* was ubiquitously expressed in the neural folds of E8.5 embryos (n=8) as shown on whole embryos in lateral view (Q), frontal view (R) and back view (S). Whole mount X-Gal stained *Lrp6*$^{+/-}$ embryo at E10.5 (T, lateral view) revealed that *Lrp6* continued to be expressed ubiquitously (n=12). Wild-type littermates were used as negative controls and never showed an X-Gal colour response. Scale bar: 500µm

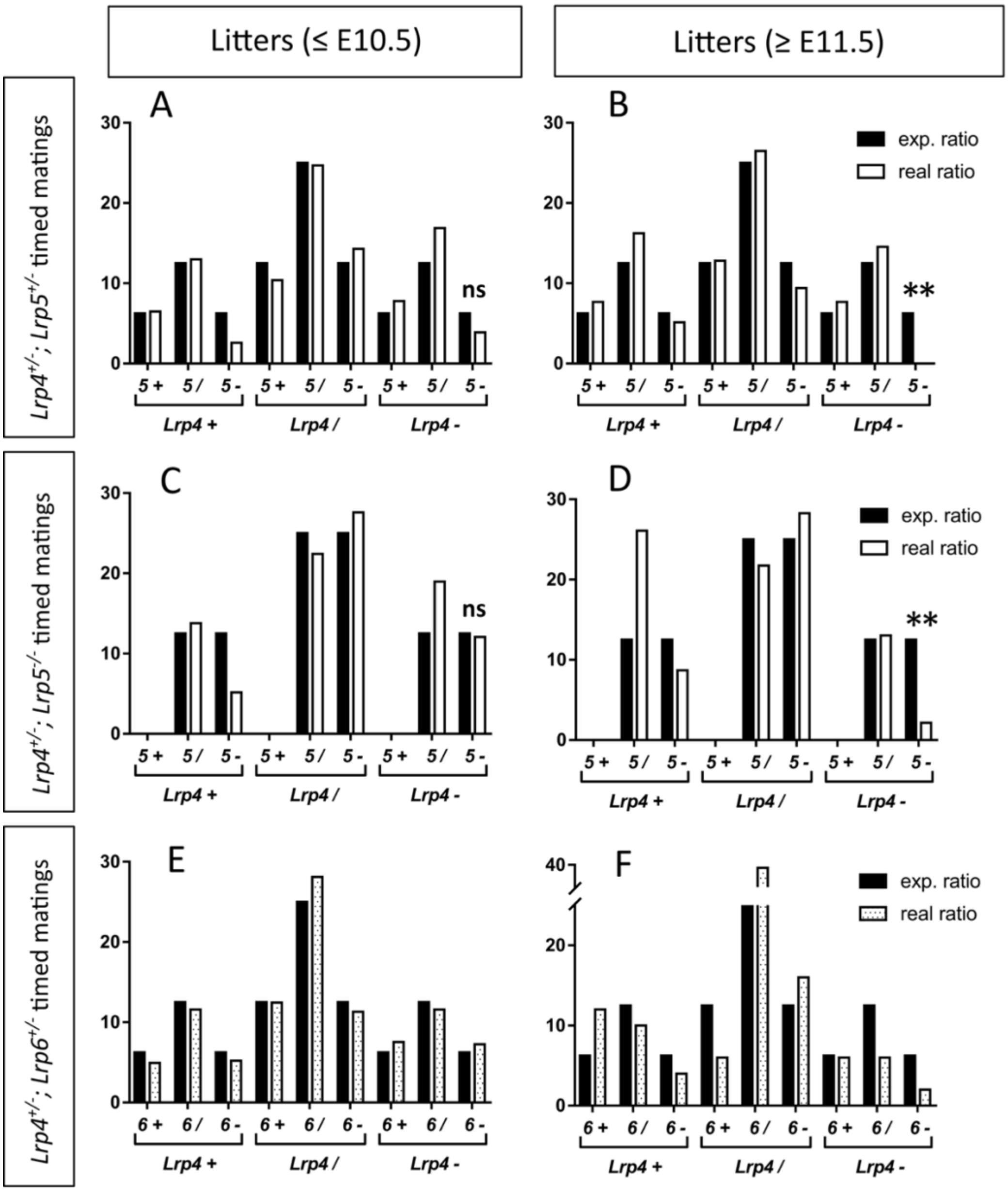

Supplementary Figure 2

***Lrp4$^{-/-}$; Lrp5$^{-/-}$* compound mutant embryos show early embryonic lethality whereas *Lrp4$^{-/-}$; Lrp6$^{-/-}$* compound mutant embryos survive throughout embryonic development**

A - B: Genotype distribution for embryos generated from *Lrp4$^{+/-}$; Lrp5$^{+/-}$* x *Lrp4$^{+/-}$; Lrp5$^{+/-}$* timed matings. **A:** At E9.5 and E10.5 there was no significant difference to the expected ratio for *Lrp4$^{-/-}$; Lrp5$^{-/-}$* embryos (one sample t-test: n.s. with p-value = 0,5183, n = 77). **B**: At later developmental stages (> E11.5) *Lrp4$^{-/-}$; Lrp5$^{-/-}$* compound mutants could no longer be detected (one sample t-test: ** with p-value = 0,0061, n = 117). x axis labelling for the different genotypes: + indicates +/+, / indicates +/- and - indicates -/- genotype.

C - D: Genotype distribution for embryos generated from *Lrp4$^{+/-}$;* ***Lrp5$^{-/-}$*** x *Lrp4$^{+/-}$; Lrp5$^{+/-}$* timed matings confirmed embryonic lethality of *Lrp4$^{-/-}$; Lrp5$^{-/-}$* compound mutants after E11.5. [D: n.s. with p-value = 0,5898, n=136. **C:** n.s. with p-value = 0,9475, n = 90. **D:** ** with p-value = 0,0009, n = 46 (for rate of *Lrp4$^{-/-}$; Lrp5$^{-/-}$* in D - F the one sample t-test was used)].

E: Genotype distribution of embryos at E9.5 and E10.5 generated from *Lrp4*$^{+/-}$*; Lrp6*$^{+/-}$ x *Lrp4*$^{+/-}$*; Lrp6*$^{+/-}$ timed matings showed no significant differences to the expected Mendelian ratios (one sample t-test: n.s. with p-value = 0,6134, n = 345). **F:** Genotype distribution of embryos at E 11.5 and older generated from *Lrp4*$^{+/-}$*; Lrp6*$^{+/-}$ x *Lrp4*$^{+/-}$*; Lrp6*$^{+/-}$ timed matings showed slightly decreased but no significant differences to the expected Mendelian ratios of *Lrp4*$^{-/-}$*; Lrp6*$^{-/-}$ compound mutants [(3.34% versus 6.25% expected ratio) (one sample t-test: n.s. with p-value = 0,714, n = 60).

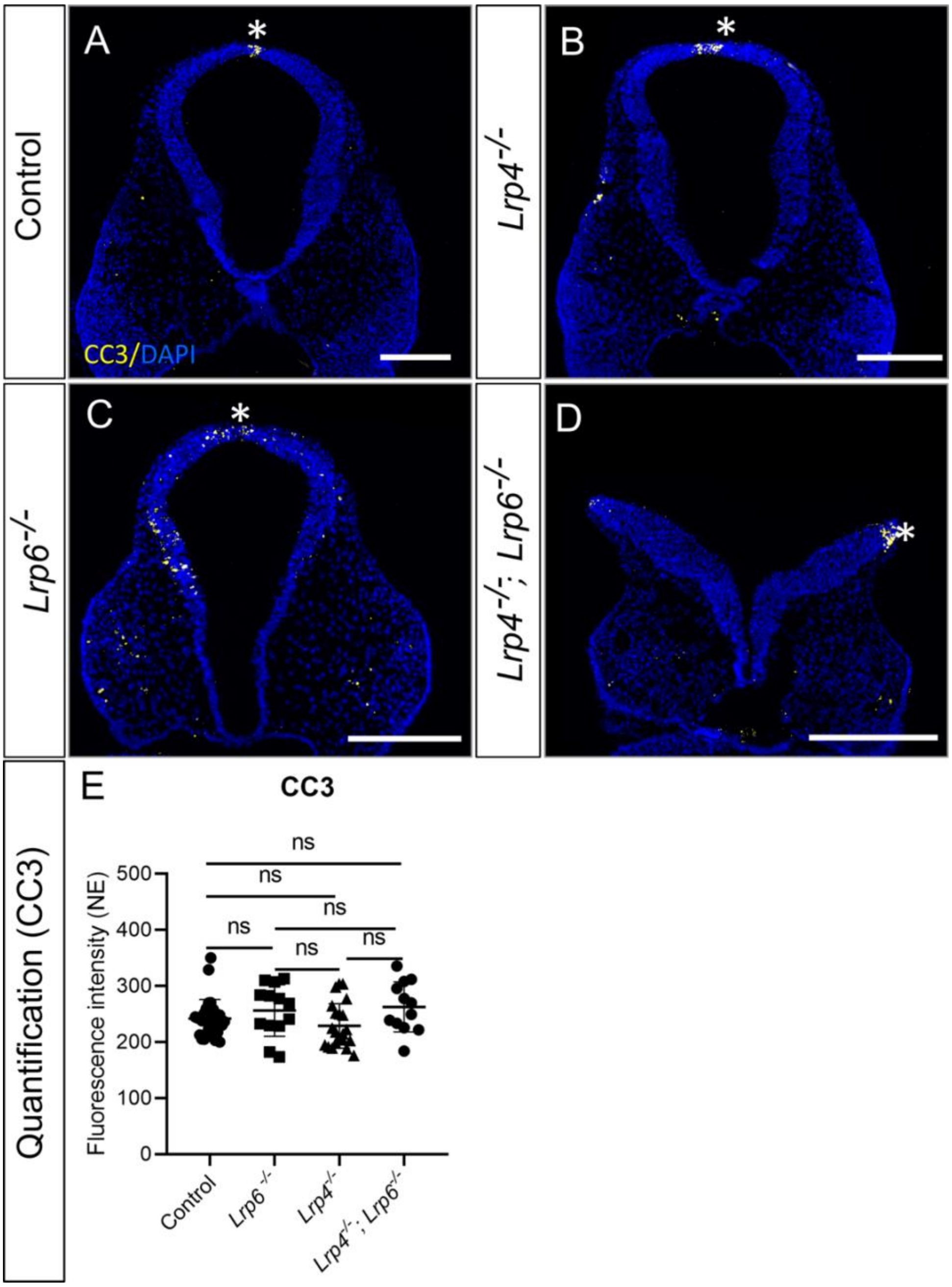

Supplementary Figure 3

Pattern of apoptosis in all *Lrp* genotypes was comparable to wild-type controls at E 9.5

Immunohistology staining, detecting cleaved-Caspase-3 (CC3) staining on coronal forebrain sections, highlights clusters of apoptotic cells in the genuine apoptosis site within the dorsal midline (as indicated by the asterisks) of controls (n = 3), *Lrp4*$^{-/-}$ (n = 3), *Lrp6*$^{-/-}$ (n = 4) and *Lrp4*$^{-/-}$; *Lrp6*$^{-/-}$ compound mutant embryos (n = 3). Sporadic clusters of apoptotic cells in the lateral domain close to the optic cup of *Lrp6*$^{-/-}$ forebrains did not lead to an overall significant increase in numbers of CC3 positive cells. No significant differences were detected between genotypes. Graph shows quantification of CC3 immunohistochemistry signal intensity (individual data points represent measurements on individual sections), y axis: mean fluorescence intensity in the entire neuroepithelium. A total of 4 to 9 coronal sections from each embryo were examined. Scatter plot presents mean ± s.d.; the significance was assessed with one-way ANOVA; scalebars: 200 µm

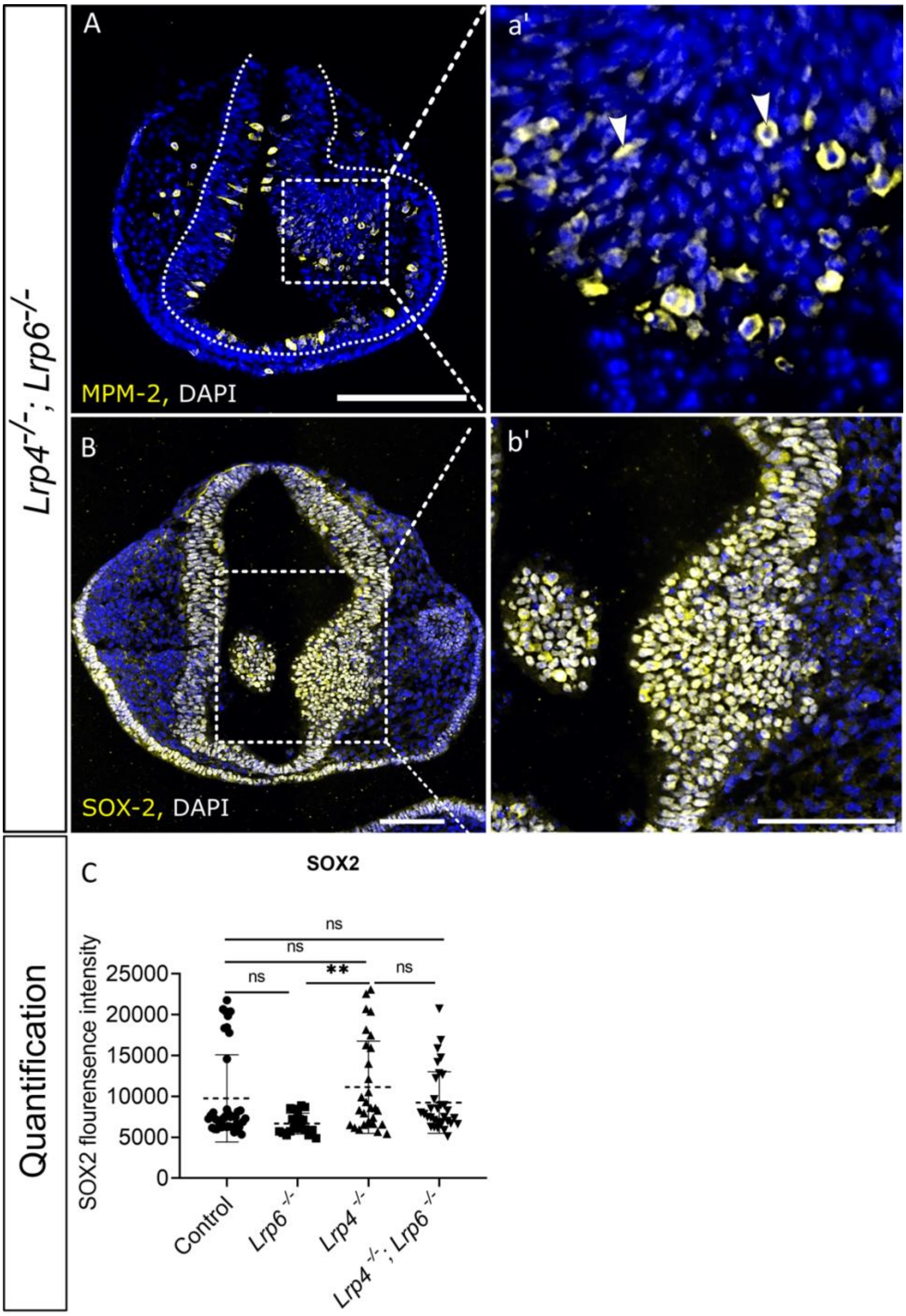

Supplementary Figure 4:

***Lrp4*$^{-/-}$; *Lrp6*$^{-/-}$ compound mutants developed excrescences in the forebrain neuroepithelium**

A: Coronal forebrain sections of *Lrp4*$^{-/-}$; *Lrp6*$^{-/-}$embryos at E9.5 show MPM-2 staining. **a':** Inset displays (4x) magnified view of MPM-2 staining in neuroepithelial excrescences (arrowheads) indicating higher mitotic activity in these areas. Excrescences showed aberrant cellular organization of the neuroepithelium. Scale bar: 200µm. **B:** Coronal forebrain sections of *Lrp4*$^{-/-}$; *Lrp6*$^{-/-}$embryos at E9.5 show immunostaining for neural stem cell marker SOX-2. **b':** Inset displays (2x) magnified view of SOX-2 staining in neuroepithelial excrescences. Cells within neuroepithelial excrescences were SOX2 positive and therefore retained their progenitor character. Scale bar: 100µm.

C: Quantification of SOX2 immunofluorescence intensity in the neuroepithelium of control embryos (n = 4), *Lrp6*$^{-/-}$ embryos (n = 3), *Lrp4*$^{-/-}$ embryos (n = 3), and *Lrp4*$^{-/-}$; *Lrp6*$^{-/-}$embryos (n= 4). Overall SOX2 levels did not show significant differences between genotypes, except higher SOX2 levels in *Lrp4*$^{-/-}$ embryos compared to *Lrp6*$^{-/-}$ embryos. A total of 7 - 12 coronal sections from each embryo were examined. Scatter plot presents mean ± s.d.; the significance was assessed with one-way ANOVA; p value: ** $p < 0.01$.

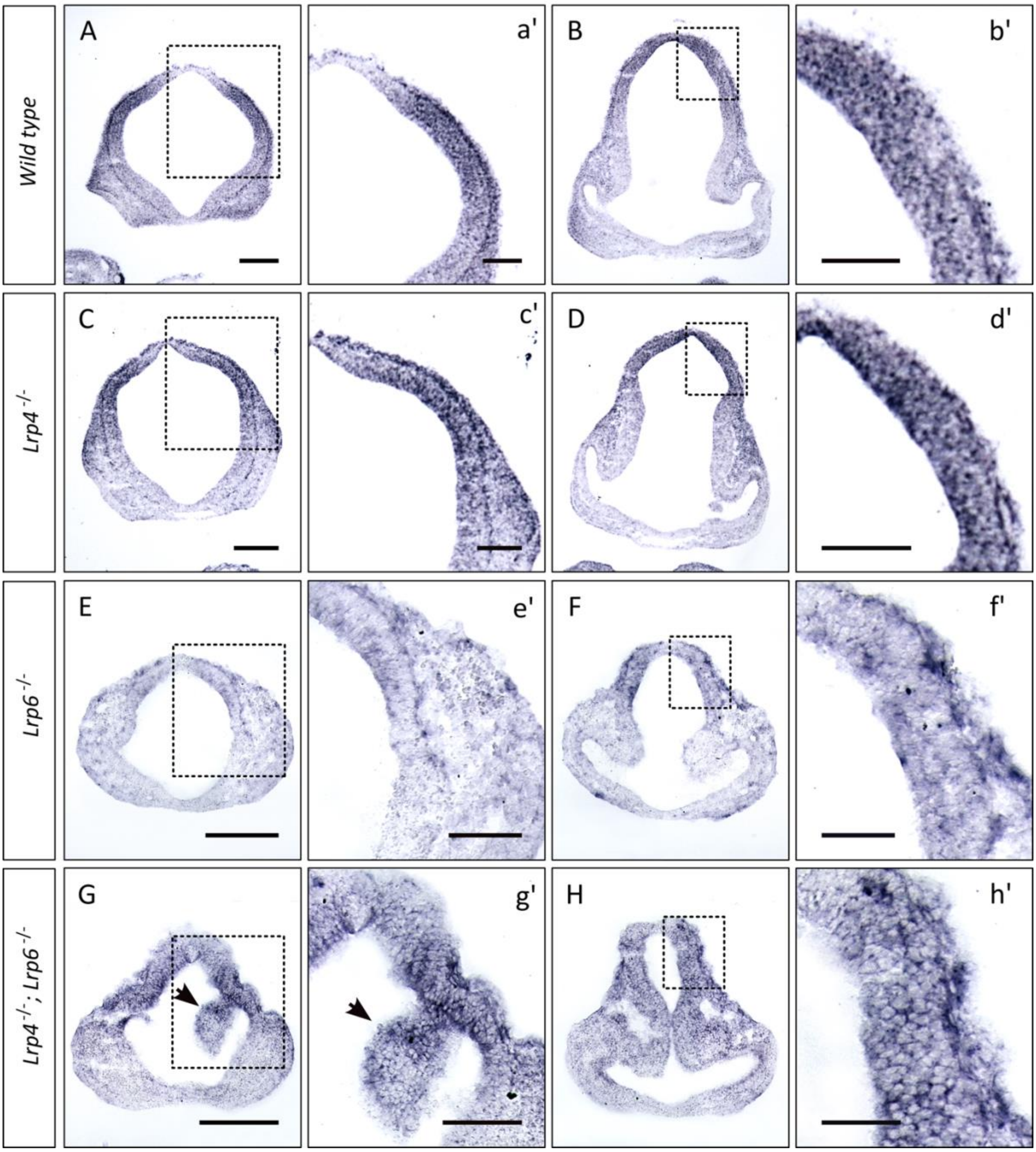

Supplementary Figure 5

Expression of *Lef1*, a key target and mediator of the WNT/β-catenin signalling pathway

ISH for *Lef1* on forebrain sections from two different planes **A, B**: *Lef1* was expressed in the dorsal lateral region of the developing forebrain of control embryos. **C, D**: Expression of *Lef1* was not altered in *Lrp4*$^{-/-}$ embryos (n=4). **E, F:** LRP6-deficient embryos (n=5) displayed a great reduction of *Lef1* expression in the neuroepithelium. **G, H**: *Lrp4*$^{-/-}$; *Lrp6*$^{-/-}$ compound mutant embryos (n=3) showed elevated levels of *Lef1* transcripts compared to *Lrp6*$^{-/-}$ embryos. *Lef1* was expressed in neuroepithelial excrescences of *Lrp4*$^{-/-}$; *Lrp6*$^{-/-}$ compound mutant embryos (arrowheads in G and g'). a', c', e' and g' insets of boxed areas are 2x magnified, scale bars: 100µm. b', d', f', and h' insets of boxed areas are 4x magnified, scale bars: 50µm. A, C, E, G, scale bars: 200µm.
